# Real-world Validation of TMB and Microsatellite Instability as Predictive Biomarkers of Immune Checkpoint Inhibitor Effectiveness in Advanced Gastroesophageal Cancer

**DOI:** 10.1158/2767-9764.CRC-22-0161

**Published:** 2022-09-21

**Authors:** Ryon P. Graf, Virginia Fisher, James Creeden, Alexa B. Schrock, Jeffrey S. Ross, Halla Nimeiri, Geoffrey R. Oxnard, Samuel J. Klempner

**Affiliations:** 1 Foundation Medicine, Cambridge, Massachusetts.; 2 Upstate Medical University, Syracuse, New York.; 3 Department of Medicine, Division of Hematology-Oncology, Massachusetts General Hospital, Boston, Massachusetts.

## Abstract

Patients with advanced gastroesophageal cancer (mEG) and tumor mutational burden ≥10 mut/Mb (TMB ≥ 10) have more favorable outcomes on immune checkpoint inhibitor (ICPI) monotherapy compared with chemotherapy in subgroup analyses of randomized controlled trials. We sought to evaluate the robustness of these associations in real-world settings where patients and practices are more diverse. A total of 362 2 L and 692 1 L patients, respectively received ICPI (*n* = 99, 33) or chemotherapy (*n* = 263, 659) across approximately 280 U.S. academic or community-based cancer clinics March 2014–July 2021. Deidentified data were captured into a real-world clinico-genomic database. All patients underwent Foundation Medicine testing. Time to next treatment (TTNT) and overall survival (OS) comparing ICPI versus chemotherapy were adjusted for treatment assignment imbalances using propensity scores. 2L: TMB ≥ 10 had more favorable TTNT [median 24 vs. 4.1 months; HR: 0.19; 95% confidence interval (CI): 0.09–0.44; *P* = 0.0001] and OS (median 43.1 vs. 6.2 months; HR: 0.24; 95% CI: 0.011–0.54; *P* = 0.0005), TMB < 10 did not (*P* > 0.05). 1L: TMB ≥ 10 had more favorable TTNT (not reached vs. median 4.1 months; HR: 0.13; 95% CI: 0.03–0.48; *P* = 0.0024) and OS (not reached vs. median 17.1 months; HR: 0.30; 95% CI: 0.08–1.14; *P* = 0.078), TMB < 10 had less favorable TTNT (median 2.8 vs. 6.5 months; HR: 2.36; 95% CI: 1.25–4.45; *P* = 0.008) and OS (median 4.5 vs. 13.1 months; HR: 1.82, 95% CI: 0.87–3.81; *P* = 0.11). TMB ≥ 10 robustly identifies patients with mEG with more favorable outcomes on 2 L ICPI monotherapy versus chemotherapy. 1 L data are more limited, but effects are consistent with 2L.

**Significance:**

Using real-world data, we sought to evaluate robustness of these clinical associations using the same assay platform and biomarker cut-off point used in both clinical trials and pan-tumor CDx approvals for later treatment lines. TMB ≥ 10 robustly identified patients with mEG with more favorable outcomes on ICPI monotherapy versus chemotherapy and suggests this subset of patients could be targeted for further trial development.

## Introduction

The treatment landscape of gastroesophageal adenocarcinoma has rapidly evolved in the last several years, with immune checkpoint inhibitors (ICPI) becoming an integral component of standard therapies. The recent randomized first-line phase III trial CheckMate-649 demonstrated that the addition of nivolumab to chemotherapy improved progression-free survival (PFS) and overall survival (OS) in advanced gastroesophageal adenocarcinomas (1). The frontline Keynote-590 study similarly showed that first-line pembrolizumab plus chemotherapy was superior to chemotherapy in esophageal cancers (2).

The Keynote-062 study paradoxically did not show superiority of adding pembrolizumab to chemotherapy but did hit a prespecified noninferiority margin comparing first-line ICPI without chemotherapy versus chemotherapy (3). While these results in aggregate represent substantial additions to treatment options, there remains much uncertainty about when single-agent ICPI without chemotherapy might be favorable, or when immunotherapy might have a low probability of benefit.

While higher PD-L1 levels identify patients with favorable outcomes with ICPI-containing regimens compared with chemotherapy alone in all of these studies, concerns around spatial and temporal PD-L1 heterogeneity support a need for more rigorous assessments with other candidate biomarkers, such as tumor mutational burden (TMB) and microsatellite instability high (MSI-H; ref. 4). Currently, the National Comprehensive Cancer Network does not endorse single-agent ICPI without chemotherapy as an option in first-line settings. ICPI monotherapy is listed as an option for the second-line and beyond (5) for patients who are mismatch repair deficient or MSI-H (6) or have TMB ≥ 10 mutations/megabase (mut/Mb; ref. 7).

Biomarkers with the greatest clinical utility predict the relative benefit of one therapy compared with another for subgroups of patients testing both positive or negative (or high or low) for the biomarker (8). While pembrolizumab failed to show superiority to paclitaxel in the intent-to-treat (ITT) population of the phase III randomized second-line Keynote-061 study (9), a *post hoc* subgroup analysis reported superior PFS [HR: 0.69; 95% confidence interval (CI): 0.31–1.51] and OS (HR: 0.34; 95% CI: 0.14–0.83) for ICPI versus chemotherapy in the group with high TMB, while the group with low TMB had comparable to worse PFS (HR: 1.46; 95% CI: 1.07–1.99) and OS (HR: 0.98; 95% CI: 0.71–1.35; ref. 10). Similarly, *post hoc* subgroup analysis for KN-062 reported superior PFS (HR: 0.52; 95% CI: 0.24–1.13) and OS (HR: 0.34; 95% CI: 0.14–0.82) for ICPI versus chemotherapy in the group with high TMB, while the group with low TMB had comparable to worse PFS (HR: 1.73; 95% CI: 1.26–2.38) and OS (HR: 1.41; 95% CI: 1.02–1.95) when treated with pembrolizumab (11).

Patients who enroll in clinical trials are often healthier and have better socioeconomic status compared to the overall patient population (12), raising the importance of drug and biomarker effectiveness evaluations reflecting broader real-world patient populations and treatment practices (13). For these reasons, we sought to compare the outcomes of real-world patients on ICPI monotherapy versus chemotherapy stratified by biomarkers. We focused primarily on TMB, given existing predictive clinical validity (14) and cross-platform harmonizations (15), using the same platform (Foundation Medicine) and cutoff of 10 mut/Mb as the existing FDA CDx for pembrolizumab (14).

## Materials and Methods

### Real-world Analysis Design

Observational data analyses require special considerations when assessing the validity of biomarkers or effectiveness of drugs. To this end, we employed two complementary techniques: propensity analyses and crossover analyses, with interpretations resting on consistency of observations across different cohorts and methods of evaluation, similar to prior real-world analyses in metastatic prostate cancer (16).

In randomized controlled trials (RCT), treatment assignments are randomized among otherwise homogeneous patient groups, ensuring that between-group differences represent causal effects of treatment. In real-world settings, the treatment assignment is at the discretion of the treating physician who uses clinical factors and best judgment to guide treatment choice. Causal inference approaches (using techniques such as propensity scores) explicitly adjust imbalances in the clinical and pathological factors that influence these decisions by variably weighting or excluding patients from analyses (17). Causal inference enables direct comparison of drug effectiveness in biomarker-defined groups of patients in specific clinical scenarios, but is dependent upon careful adjustment of treatment assignment biases, and cannot adjust for unquantifiable factors influencing treatment decisions or unknown confounders (17). Crossover analyses comparing the drug effectiveness within the same patient, such as when Drug B immediately follows Drug A, overcome these caveats, but at the cost of losing some clinical context and likely bias of disadvantage to Drug B, and preventing OS analyses. Evaluating biomarker-identified populations with potential enhanced effectiveness of Drug B relative to Drug A are valid, but not the opposite. Statistical Analysis and Interpretations (see below) make use of both causal inference and crossover analyses in tandem.

**FIGURE 1:**
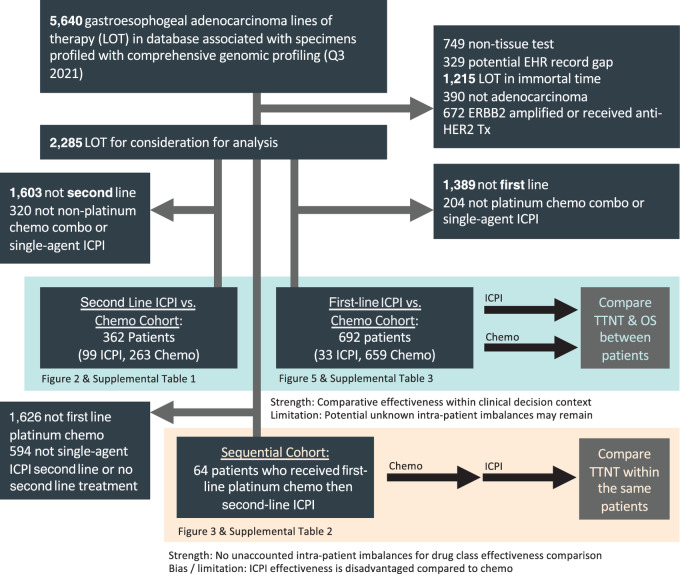
Cohort diagram. Cohort selection diagrams and analysis overviews.

We first evaluated ICPI versus chemotherapy effectiveness between patients in second-line settings, stratified by TMB, then evaluated whether patients with TMB ≥ 10 mut/Mb had enhanced relative effectiveness of second-line ICPI when used after first-line chemotherapy within the same patients. Finally, we evaluated the outcomes of patients who received first-line ICPI in comparison with standard first-line chemotherapy (see Fig. 1 for cohort overviews).

### Patient Selection

The study comprised patients with confirmed diagnosis of gastroesophageal adenocarcinoma (gastric, esophageal, or gastroesophageal junction) included in the U.S.-wide Flatiron Health-Foundation Medicine (FMI) deidentified clinico-genomic database between January 2011 and June 2021. All patients underwent genomic testing using Foundation Medicine comprehensive genomic profiling (CGP) assays. Deidentified clinical data originated from approximately 280 U.S. cancer clinics (∼800 sites of care). Retrospective longitudinal clinical data were derived from electronic health records (EHR), comprising patient-level structured and unstructured data, curated via technology-enabled abstraction of clinical notes and radiology and/or pathology reports, which were linked to genomic data derived from Foundation Medicine testing by deidentified, deterministic matching (18). Clinical data included demographics, clinical and laboratory features, timing of treatment exposure, and survival.

Patient records were included in this study if they received a first- or second-line single-agent anti-PD1 or anti-PD-L1 agent or standard chemotherapy (platinum regimens for first-line, non-platinum regimens for second line) in the metastatic setting and had TMB assessed via a tissue specimen. Patients who received both ICPI and chemotherapy in combination at the same time were not included because of low numbers. Patients must have additionally tested negative for *ERBB2* amplification via CGP and not have received an anti-HER2 agent. Analyses were conducted in three cohorts (Fig. 1):

2 L Comparative Effectiveness Cohort: Patients who received a single-agent ICPI or non-platinum chemotherapy in second line.

Sequential Cohort: Patients who received platinum chemotherapy in first line, and received a single-agent ICPI in second line.

1 L Comparative Effectiveness Cohort: Patients who received either a single-agent ICPI, or a platinum-containing chemotherapy regimen in the first line.

Institutional Review Board approval of the study protocol was obtained prior to study conduct and included a waiver of informed consent.

### Comprehensive Genomic Profiling

Hybrid capture-based next-generation sequencing (NGS) assays were performed on patient tumor specimens in Clinical Laboratory Improvement Amendments–certified, College of American Pathologists–accredited laboratory (Foundation Medicine, Cambridge, MA). Samples were evaluated for alterations as described previously (19). TMB was determined on up to 1.1 Mb of sequenced DNA (20). MSI status was determined on 95–114 loci, as described previously (21).

### Outcomes

Time to next treatment (TTNT), like PFS, is a time-to-event proxy for drug clinical effectiveness (22). TTNT was calculated from treatment start date until the start of next treatment line (due to any cause), or death. Patients not yet reaching next treatment line or death were right censored at date of last clinical visit, laboratory result, or medication order. OS was calculated from start of treatment to death from any cause, and patients with no record of mortality were right censored at the date of last clinic visit or structured activity. Because patients cannot enter the database until a CGP report is delivered, OS risk intervals were left truncated to the date of CGP report to account for immortal time (23, 24). Flatiron Health database mortality information is a composite derived from three sources: documents within the EHR, Social Security Death Index, and a commercial death dataset mining data from obituaries and funeral homes. This mortality information has been externally validated in comparison to the National Death Index with >90% accuracy (25).

### Statistical Analysis and Interpretations

A prospectively declared statistical analysis plan was developed and executed. Consistent with ISPOR guidelines (26), the inclusion criteria, exclusion criteria, potential biases, primary outcome measures, exploratory outcome measures, handling of missing data, and all methods described below were prospectively specified prior to analysis execution unless otherwise noted. The prespecified analysis compared the effectiveness of ICPI versus chemotherapy in patients stratified by TMB < 10 mut/Mb and TMB ≥ 10 mut/Mb. Stratification by MSI status and PD-L1 status was included as a secondary analysis. Stratification by biomarkers other than TMB, MSI, and PD-L1 were not prespecified.

Differences in time-to-event outcomes were assessed with the log-rank test and Cox proportional hazard (PH) models. *χ*^2^ tests and Wilcoxon rank-sum tests were used to assess differences between groups of categorical and continuous variables, respectively. Multiple comparison adjustments were not performed; *P* values are reported to quantify the strength of association for biomarker and each outcome, not for null hypothesis significance testing, and interpretations are adopted broadly considering consistency of multiple outcome measures in concert (TTNT, OS) across defined cohorts (interpatient vs. intrapatient) with no outcome measure or cohort standing on its own. The default interpretation is that a biomarker correlating with OS but not TTNT within a cohort is likely a confounding artifact, and a biomarker correlating with TTNT but not OS is not remarkable. In addition, while the effect size estimates may vary by cohort, the default assumption is that a biomarker effect should not be specific to any of the cohorts evaluated. Missing values were handled by simple imputation with expected values determined using random forests with the R package “missForest.” In subsequent analyses, imputed values were treated identically to measured values.

Propensity analyses made use of inverse probability of treatment weights targeting the average treatment effect in the ICPI-treated population, implemented with R package “MatchIt.” Weights were capped at 10 equivalents to limit influence per observation. Among patients receiving chemotherapy, those with characteristics most similar to the ICPI patient population were weighted more, and those less like the ICPI patients weighted less. These weights were included in all Kaplan–Meier visualizations and Cox PH models, unless otherwise noted. Available features related to treatment assignment of ICPI versus chemotherapy included for adjustment in propensity models were: Eastern Cooperative Oncology Group (ECOG; 0–2 vs. 3+), abnormal labs (bilirubin above upper limit of normal [ULN], albumin below ULN, or creatinine above ULN), PD-L1 [combined positive score (CPS) ≥ 5 vs. not], stage at diagnosis (stage IV vs. not), prior surgery (yes vs. no) and TMB (continuous). Standardized mean difference (SMD) was utilized to assess balance, and within 10% considered acceptable (27).

To focus on the 10 mut/Mb threshold, propensity weights were created separately for TMB ≥ 10 group (TMB-high) and TMB < 10 (TMB-low) group for best possible within-group balance. Predictive biomarker associations (28) made use of inverse-propensity weighted multivariable Cox proportional hazards regression models containing at minimum the following variables: drug class (ICPI or taxane), TMB (high vs. low), and the interaction term between drug class and biomarker. Models evaluating intra-patient treatment interactions in the Sequential Cohort were additionally clustered on the individual patient, making use of robust variances calculated by generalized estimating equations within a working independence structure. HRs were then generated from adjusted Cox models stratified by group (i.e., TMB high vs. low). R version 3.6.3 software was used for all statistical analyses.

Non-proportional hazards over time between treatment groups can limit the interpretability of the HR as an effect measure. For this reason, prespecified methods of HR estimates were augmented with analyses of 3-year restricted mean survival times (29, 30)) calculated as the area under the stratum-specific Kaplan–Meier curves at 1-month intervals up to 36 months. In an effort to assess the contribution of TMB in microsatellite stable (mss) tumors, we performed an additional exploratory analysis combining patients from the 1 L and 2 L Comparative Effectiveness Cohorts, limiting to those testing MSS only. For this analysis, an adjusted HR was generated per subgroup that contained adjustment for prognostic factors including line of therapy (1 L vs. 2L), age, ECOG (0–2 vs. 3+), abnormal labs (bilirubin above ULN, albumin below ULN, or Creatinine above ULN), stage at diagnosis (stage IV vs. not), and body mass index (BMI).

### Data Availability

The data supporting the findings of this study originated from Flatiron Health, Inc. and Foundation Medicine, Inc. These deidentified data may be made available upon request, and are subject to a license agreement with Flatiron Health and Foundation Medicine; interested researchers should contact <cgdb-fmi@flatiron.com> and <dataaccess@flatiron.com> to determine licensing terms.

## Results

### Characteristics of Analysis Cohorts

2 L Comparative Effectiveness Cohort: After selection 263 patients received 2 L non-platinum chemotherapy, and 99 patients received 2 L ICPI (Fig. 1; Supplementary Table S1). No differences with *P* < 0.05 were observed by treatment group for age, sex, stage at diagnosis, smoking status, prior surgery, ECOG, albumin, or bilirubin. However, patients receiving 2 L ICPI had higher TMB (*P* < 0.001), PD-L1 CPS scores (*P* < 0.001), and more frequent MSI-H (*P* < 0.001). The median TMB of the cohort was 3.8, with interquartile range of 1.7–6.3 (Supplementary Table S1), and is consistent with prior reports in this population (31).

Sequential Cohort: After selection, 64 patients received 1 L platinum chemotherapy followed by 2 L ICPI (Fig. 1; Supplementary Table S2). Of these, 17 had TMB ≥ 10 and 47 had TMB < 10. Differences of *P* < 0.05 were not observed by TMB group for age, sex, stage at diagnosis, smoking status, prior surgery, ECOG, albumin, bilirubin, or PD-L1. However, TMB and MSI were highly correlated (*P* < 0.001); of the TMB ≥ 10 group, 14 had MSI-H, 2 had MSS and 1 had unknown MSI status. Among the TMB < 10 group, 0 were MSI-H, 36 were MSS, and 11 were MSI unknown. The median TMB of the cohort was 4.7, with interquartile range of 2.5–10.6 (Supplementary Table S2).

1 L Comparative Effectiveness Cohort: After selection, 659 patients received 1 L platinum chemotherapy, and 33 patients received first-line ICPI monotherapy (Fig. 1; Supplementary Table S3). Compared with patients receiving chemotherapy, patients receiving ICPI monotherapy were older (median 70 vs. 66; *P* = 0.038), less likely to be stage IV at diagnosis (27.3% vs. 66.5%; *P* < 0.001), more likely to have had prior surgery (69.7% vs. 20%; *P* < 0.001), and more likely to have PD-L1 testing available (*P* < 0.001). The median TMB of the cohort was 3.8, with interquartile range of 1.7–6.3 (Supplementary Table S3). Additional breakdown of TMB per cohort and by MSI and PD-L1 status can be found in Supplementary Tables 4–6.

### Real-world Patients Receiving Second-line ICPI Monotherapy Versus Chemotherapy Have More Favorable Outcomes When TMB ≥ 10 but not TMB < 10

In the TMB < 10 subgroup, all features had SMD < 10% after weighting, with the exception of a bias toward higher TMB among patients receiving ICPI (Supplementary Fig. S1A). In the TMB ≥ 10 subgroup, while imbalances were greatly reduced, residual imbalances of SMD ≥ 10% were present, such that patients receiving ICPI were less likely to have had surgery, and more likely to be stage IV at diagnosis (Supplementary Fig. S1B–D).

**FIGURE 2:**
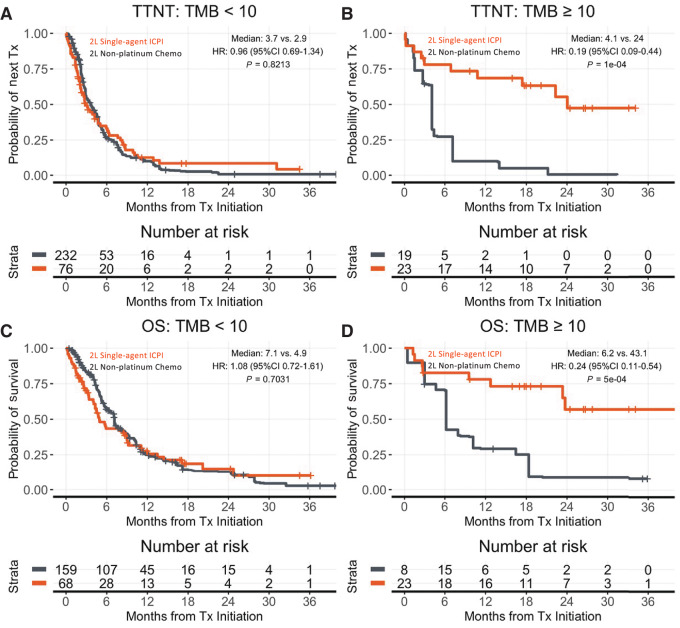
Adjusting for known treatment assignment imbalances, patients receiving second-line (2L) ICPI versus chemotherapy have more favorable outcomes when TMB ≥ 10 but not TMB < 10. Kaplan–Meier curves are adjusted for imbalances (propensity weights applied). TTNT is shown by drug class for TMB < 10 (**A**), and TMB ≥ 10 (**B**). OS is shown by drug class for TMB < 10 (**C**), and TMB ≥ 10 (**D**). *x*-axis is truncated at 36 months. OS estimates are left truncated to reflect delayed entry to at-risk table (see Materials and Methods). Visualizations are adjusted by propensity weights. Analyses unadjusted for propensity weights have similar results (see Supplementary Fig. S2). Interaction terms in interaction models (see Materials and Methods) for TTNT and OS, respectively. *P* < 0.0001 and 0.0028 (for full models, see Supplementary Fig. S4).

Among patients treated with single-agent ICPI versus non-platinum chemotherapy in 2 L (after prior platinum chemotherapy in first line), those with TMB < 10 (*n* = 76 ICPI, 244 chemotherapy) had comparable TTNT (median 2.9 vs. 3.7 months; HR: 0.96; 95% CI: 0.69–1.34; *P* = 0.82) and OS (median 4.9 vs. 7.1 months; HR: 1.08; 95% CI: 0.72–21.6; *P* = 0.70). However, patients with TMB ≥ 10 (*n* = 23 ICPI, 19 chemotherapy) had more favorable TTNT (median 24 vs. 4.1 months; HR: 0.19; 95% CI: 0.09–0.44; *P* = 0.0001) and OS (median 43.1 vs. 6.2 months; HR: 0.24; 95% CI: 0.011–0.54; *P* = 0.0005; Fig. 2; Supplementary Fig. S2 and S3). Sensitivity analyses unadjusted for imbalances show similar results (Supplementary Fig. S4). RSMT analyses additionally show similar results (Supplementary Table S7).

### Patients Receiving First-line Chemotherapy Followed by Second-line ICPI Had Longer TTNT on Second-line ICPI Compared with First-line Chemotherapy When TMB ≥ 10 but not TMB < 10

**FIGURE 3:**
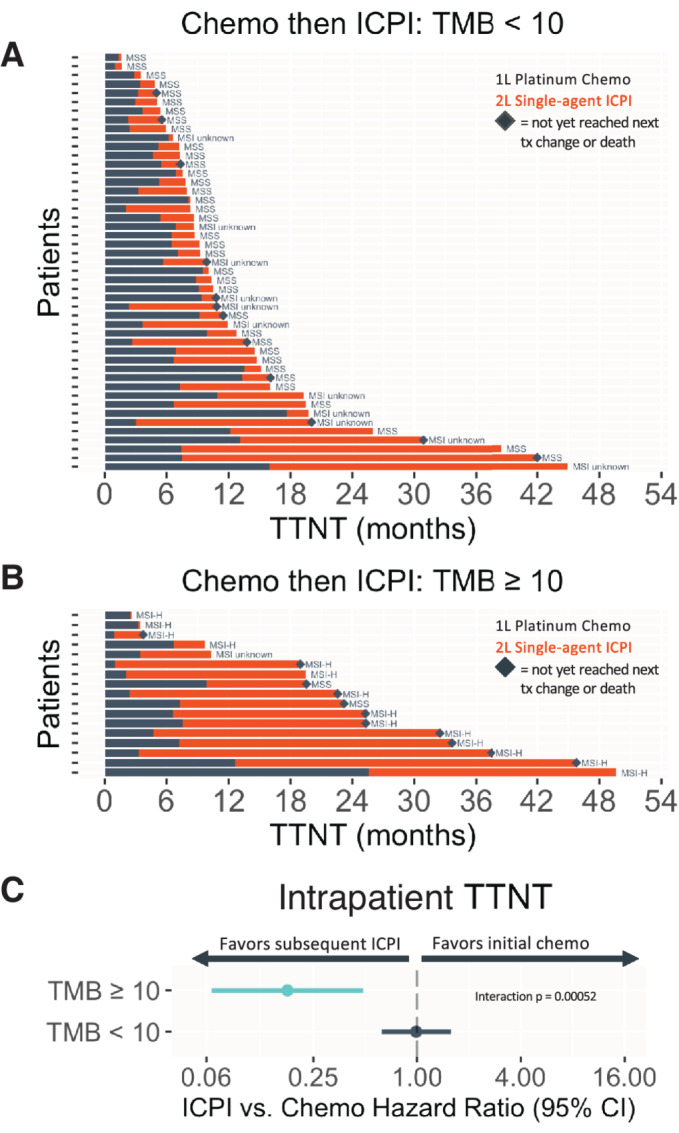
Patients receiving first-line (1L) chemotherapy followed by second-line (2L) ICPI had more favorable outcomes on second-line IPCI compared to first-line chemotherapy when TMB ≥ 10 but not TMB < 10. TTNT on first-line platinum chemotherapy and subsequent second-line ICPI are shown as stacked horizontal bar plots per patient in the Sequential Cohort for patients with TMB < 10 (**A**) and patients with TMB ≥ 10 (**B**). MSI status is listed at right hand side of bars. Point estimates and confidence intervals from Cox models comparing intrapatient TTNT are shown in **C**.

TTNT of 1 L chemotherapy are visualized from (Fig. 3A) those with TMB < 10 (*n* = 47) and (Fig. 3B) those with TMB ≥ 10 (*n* = 17), with the bars colored by the treatment line, with MSI status listed at end of the bars. MSI status was highly correlated with this TMB threshold in this cohort, with no patients with MSI-H having TMB < 10, and 14 of 17 with TMB ≥ 10 having MSI-H (2 were MSS, 1 was MSI unknown). The two with MSS and TMB ≥ 10 do not yet have record of next treatment after ICPI start (likely still benefitting from ICPI treatment), with 9.7 and 16.0 months so far. The median TTNT of second-line ICPI in the TMB < 10 group is 3.3 months (95% CI: 2.2–8.0).

Patients testing TMB < 10 did not have enrichment of benefit of either drug class (HR: 0.99; 95% CI: 0.62–1.57; *P* = 0.96). However, patients testing TMB ≥ 10 had more favorable TTNT on second-line ICPI compared with their first-line chemotherapy regimen (HR: 0.18; 95% CI: 0.064–0.48; *P* = 0.00077; Fig. 3C; Supplementary Fig. S5 and S6).

### TMB and MSI-H are Stronger Predictive Biomarkers for ICPI Monotherapy Versus Chemotherapy Benefit Than PD-L1

**FIGURE 4:**
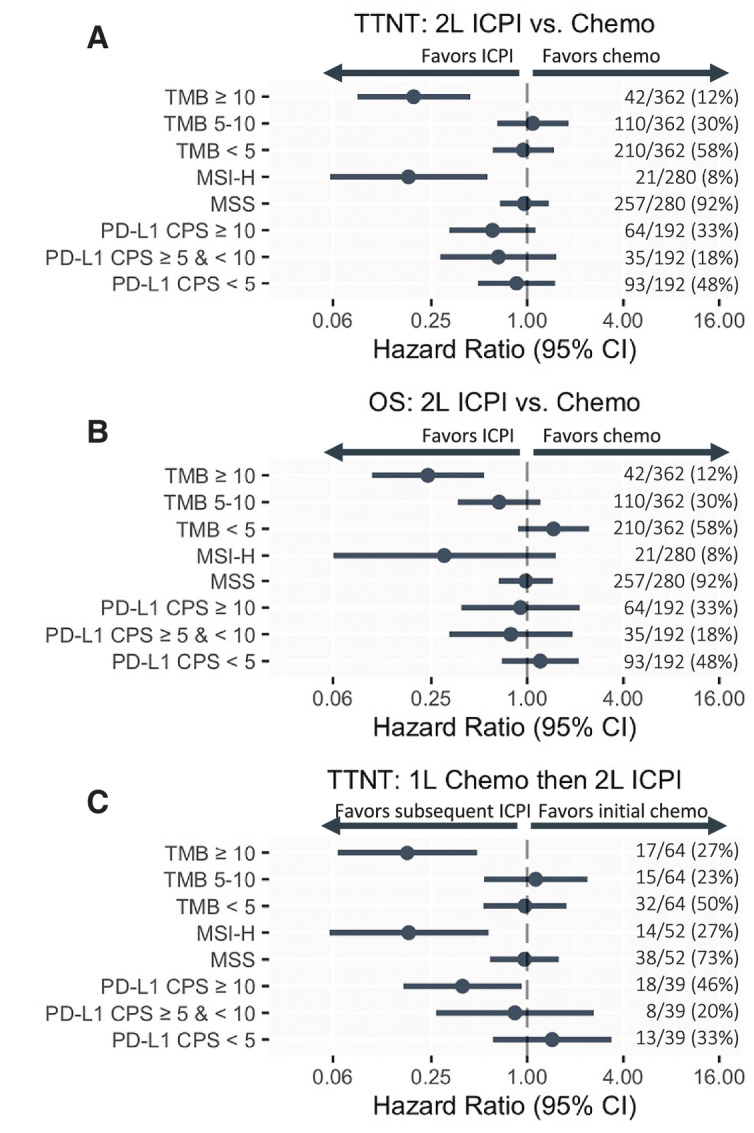
TMB and MSI-H are stronger predictive biomarkers for ICPI versus chemotherapy benefit than PD-L1. Point estimates and confidence intervals for biomarker-defined groups of patients are shown for TTNT in the second-line (2L) ICPI versus chemotherapy cohort (**A**), OS in the second-line ICPI versus chemotherapy cohort (**B**), and intrapatient TTNT (**C**). Cox multiple interaction models comparing the additive predictive associations of TMB and PD-L1 can be found in Supplementary Fig. S6.

PD-L1 CPS ≥ 5 does not have particular enrichment in patients with high TMB (Supplementary Table S8), suggesting a degree of biomarker independence in these cohorts. PD-L1 scoring was not available for 47% of the second-line comparative effectiveness cohort (Supplementary Table S1) and for 40% of the sequential cohort (Supplementary Table S2). However, the absolute number of patients with PD-L1 CPS ≥ 10 was comparable and relative prevalence of PD-L1 CPS ≥ 10 was much higher than TMB ≥ 10 or MSI-H. In the 2 L ICPI versus chemotherapy cohort as well as the sequential analysis, patients identified by TMB ≥ 10 and/or MSI-H had similar prevalence, and enrichment for observed outcomes favoring ICPI versus chemotherapy (Fig. 4A–C; Supplementary Fig. S7). We observed a modest enrichment favoring ICPI versus chemotherapy in patients identified by PD-L1 CPS ≥ 10 in TTNT of the sequential cohort, but not for TTNT or OS in the second-line comparative effectiveness cohort.

The additive predictive associations of TMB ≥ 10 and PD-L1 CPS ≥ 10 were evaluated with multivariable models containing multiple interaction terms (Supplementary Fig. S8). We did not observe a reduction in TMB ≥ 10 predictive associations with inconsistent or weak additional predictive association of PD-L1 CPS ≥ 10.

### Adjusting for Known Treatment Assignment Imbalances, Patients Receiving First-line ICPI Versus Chemotherapy Have More Favorable Outcomes When TMB ≥ 10 but not TMB < 10

In the TMB < 10 subgroup, residual imbalances remained such that patients receiving ICPI were more likely to have had surgery, and have marginally more likely to have PD-L1 CPS ≥ 5, ECOG 3 or higher, be older, or have abnormal labs. In the TMB ≥ 10 subgroup, patients receiving ICPI were marginally more likely to have been stage IV at diagnosis (Supplementary Fig. S1). However, due to only 7 first-line patients with TMB ≥ 10 receiving ICPI, results must be interpreted cautiously.

**FIGURE 5:**
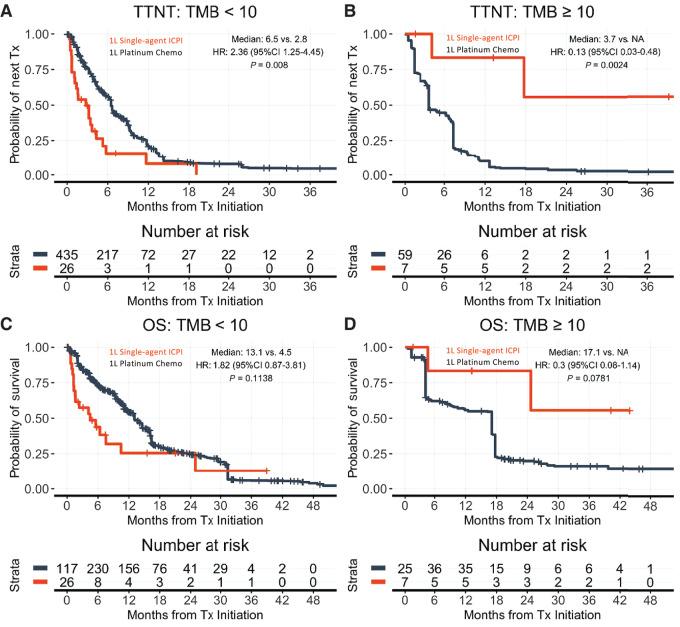
Adjusting for known treatment assignment imbalances, patients receiving first-line (1L) ICPI versus chemotherapy have more favorable outcomes when TMB ≥ 10 but not TMB < 10. Kaplan–Meier curves are adjusted for imbalances. TTNT is shown by drug class for TMB < 10 (**A**), and TMB ≥ 10 (**B**). OS is shown by drug class for TMB < 10 (**C**), and TMB ≥ 10 (**D**). *x*-axis is truncated at 36 months. OS estimates are left truncated to reflect delayed entry to at-risk table (see Materials and Methods). Visualizations are adjusted by propensity weights. Analyses unadjusted for propensity weights have similar results (see Supplementary Fig. S3). Interaction terms in interaction models (see Materials and Methods) for TTNT and OS, respectively. *P* < 0.0001 and *P* = 0.0292 (for full models, see Supplementary Fig. S7).

Among patients treated with single-agent ICPI versus non-platinum chemotherapy in first line, those with TMB < 10 (*n* = 26 ICPI, 435 chemotherapy) had inferior TTNT (median 2.8 vs. 6.5 months; HR: 2.36; 95% CI: 1.25–4.45; *P* = 0.0080) and comparable with worse OS (median 4.5 vs. 13.1 months; HR: 1.82; 95% CI: 0.87–3.81; *P* = 0.11). However, patients with TMB ≥ 10 (*n* = 7 ICPI, 59 chemotherapy) had more favorable TTNT (median not reached vs. 3.7 months; HR: 0.13; 95% CI: 0.03–0.48; *P* = 0.0024) and comparable with favorable OS (median not reached 17.1 months; HR: 0.30; 95% CI: 0.08–1.14; *P* = 0.078; Fig. 5). Sensitivity analyses unadjusted for imbalances show similar results (Supplementary Fig. S3). RSMT analyses additionally show similar results (Supplementary Table S7; Supplementary Fig. S9 and S10).

### Real-world Data Parallel RCT Data in 1L and 2L Gastroesophageal Adenocarcinomas Despite Differences in Patient Populations

**FIGURE 6:**
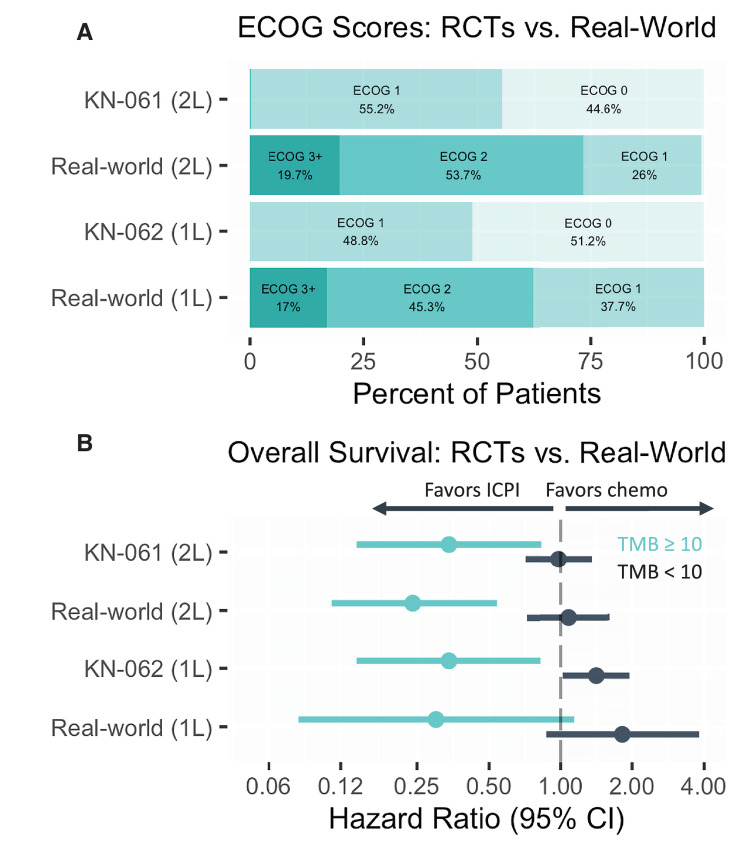
RCT and real-world cohorts have very different patient populations, similar drug-class specific TMB associations. The phase III randomized controlled trials KeyNote-061 (KN-061), KeyNote-062 (KN-062) and the second-line and first-line Comparative Effectiveness Cohorts are compared with respect to their patient ECOG scores distributions (**A**) and OS (**B**) by TMB subgroups. Notes: KN-061 had less than 1% as ECOG 2, and second-line real-world cohort less than 1% ECOG 0, these not labeled due to graphical constraints. 2 L = second line; 1 L = first line.

Using ECOG performance scores as a proxy for overall patient frailty across cohorts, the broad cohort characteristics from the phase III Keynote-061 comparing second-line pembrolizumab with paclitaxel (9), the phase III Keynote-062 first-line pembrolizumab with platinum chemotherapy (3) are compared with the second- and first-line comparative effectiveness cohorts (Fig. 6A). The OS subgroup analysis for TMB subgroups in KeyNote-061 (10), and KeyNote-062 were reported as a *post hoc* analyses (11). These subgroup analyses are shown alongside the intrapatient OS assessments of the second- and first-line comparative effectiveness cohorts (Fig. 6B). Taken together, these findings help place our observations in the context of prospective randomized data.

### Among Patients with MSS Tumors, Those with TMB ≥ 10 Have More Favorable Outcomes on ICPI Compared with Those with TMB < 10, and Comparable Outcomes with Those with TMB ≥ 10 Receiving Chemotherapy

**FIGURE 7:**
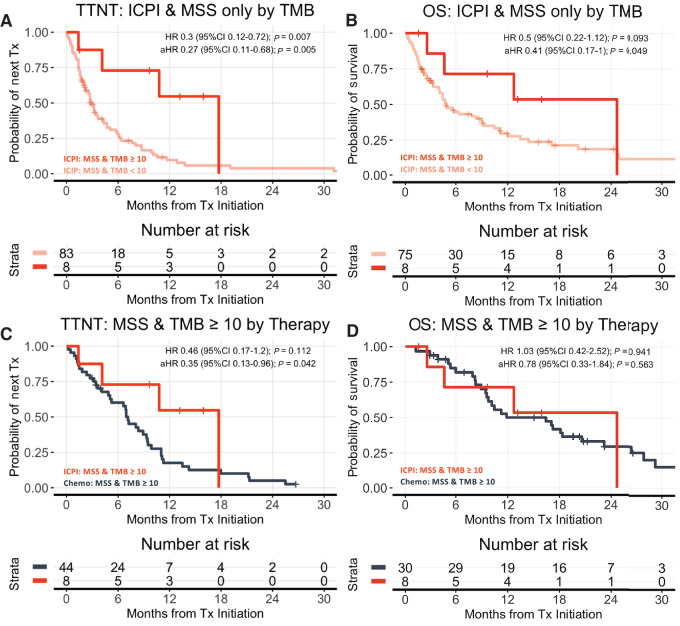
MSS only exploratory analysis. Patients testing MSS assigned ICPI from both the 1 L and 2 L comparative effectiveness cohorts were combined for an exploratory analysis, and stratified by TMB ≥ 10 for TTNT (**A**) and OS (**B**). Outcomes of patients testing both MSS and TMB ≥ 10 receiving ICPI or chemotherapy are shown for TTNT (**C**) and OS (**D**). *x*-axis is truncated at 30 months. OS estimates are left truncated (see Materials and Methods) with at-risk tables adjusted accordingly. Kaplan–Meier curves are not adjusted for propensity weights. aHR = adjusted hazard ratio, adjusted for line of therapy (1 L or 2L), age, ECOG, stage at diagnosis, abnormal laboratory values, and BMI.

Evaluating only patients with MSS tumors, and combining the 1 L and 2 L Comparative Effectiveness Cohorts, those with TMB ≥ 10 receiving ICPI (*n* = 8, 4 from 1 L cohort, 4 from 2 L cohort) had more favorable TTNT (aHR: 0.27; 95% CI: 0.11–0.68; *P* = 0.005) and OS (aHR: 0.41; 95% CI: 0.17–1.0; *P* = 0.049) compared with those with TMB < 10 receiving ICPI (*n* = 21 from 1 L cohort, 62 from 2 L cohort). Compared with those with TMB ≥ 10 receiving chemotherapy (*n* = 69 from 1 L cohort, 19 from 2 L cohort). Those with TMB ≥ 10 receiving ICPI (*n* = 8, 4 from 1 L cohort, 4 from 2 L cohort) had more favorable TTNT (aHR: 0.35; 95% CI: 0.13–0.96; *P* = 0.042) and comparable OS (aHR: 0.78; 95% CI: 0.33–1.84; *P* = 0.563; Fig. 7).

## Discussion

The treatment of gastroesophageal cancers is rapidly evolving with several recent phase III trials examining the role of ICPI added to frontline chemotherapy. Within gastric and gastroesophageal junction (GEJ) adenocarcinomas, the totality of the frontline data suggests that patients with a PD-L1 CPS of less than 5 derive no benefit from the addition of anti-PD-1 antibodies to standard chemotherapy. In fact, the European Medicines Agency has restricted the frontline nivolumab approval to patients with a PD-L1 CPS of 5 or greater. With 40%–60% of patients having a CPS of 5 or less, there will remain a large portion of ICPI naïve patients entering the second line. While the phase III KeyNote-061 second-line trial comparing paclitaxel versus pembrolizumab was overall negative, subgroup analyses suggest that high PD-L1–expressing and high TMB patients may benefit from ICPI over chemotherapy. Thus, there remains potential opportunities for ICPI development in 2 L and beyond, particularly using a biomarker-enriched approach.

The populations of patients with elevated TMB and/or high PD-L1 CPS scores are small within the published trial datasets, limiting the clinical applicability. We sought to thoroughly evaluate the clinical validity of TMB ≥ 10 by evaluating ICPI monotherapy versus chemotherapy effectiveness in biomarker-identified patients in a more diverse, real-world population of patients seen in clinical practice. We made use of two complementary approaches for comparative effectiveness that each partially overcome their respective limitations, observing consistent strong enrichment for ICPI monotherapy versus chemotherapy benefit in both interpatient and intrapatient assessments for TMB ≥ 10, as well as NGS-assessed MSI. However, we did not observe consistent results of same magnitude with PD-L1, nor consistent additional predictive value of PD-L1 to TMB. Evaluations in more diverse real-world patient populations can provide insights about current drug use that might be improved. For instance, many patients with TMB < 10 receive single-agent ICPI in real-world practice, and these patients have comparable with worse effectiveness of ICPI compared with chemotherapy (Figs. 2 and 5). While single-agent ICPI may be less physically toxic than standard generic chemotherapy (9), ICPI can be considerably more financially toxic (32), associated with quantifiably increased psychologic distress (32), and TMB might help aid sensitive treatment decisions. Taken together, our data complement the limited existing analysis and suggest TMB-high patients receiving 2 L ICPI derive significant benefit.

It has been hypothesized that effects seen with TMB ≥ 10 subgroups might be entirely explained by MSI-H. A recent *post hoc* analysis from Keynote-061 evaluating OS in TMB ≥ 10 observed that the exclusion of MSI-H patients resulted in a similar effect size comparing ICPI versus chemotherapy with wider CIs (HR: 0.38; 95% CI: 0.13–1.13; ref. 10). Of note, there are no observed TMB < 10 patients with MSI-H in our cohorts. There are only 2 MSS patients with TMB ≥ 10 in the sequential cohort, and their relative TTNT on ICPI versus chemotherapy is much greater than MSS patients with TMB < 10 (Fig. 3). Additional exploratory analyses considering only patients testing MSS, combining the 1 L and 2 L Comparative Effectiveness cohorts (Fig. 7), are consistent with enhanced effectiveness of ICPI in the MSS/TMB ≥ 10 subgroup compared with MSS/TMB < 10 receiving ICPI. However, these analyses at face value only suggest comparable or mildly more favorable outcomes of ICPI versus chemotherapy in this subgroup. Counterfactually considering the hypothesis that perhaps MSI-H alone and not merely TMB ≥ 10 might be driving observed ICPI benefit, what limited data we have does not support this hypothesis. Instead, MSI-H appears to be a phenomenon that consistently associates with high TMB, but is not the only mechanism by which elevated TMB can arise, and elevated TMB in the absence of MSI-H likely still identifies a subgroup of enhanced ICPI benefit. Whether ICPI is better than chemotherapy in the MSS/TMB ≥ 10 subgroup is best resolved in prospective trials with better defined contexts of use.

Outside MSI-H in later lines of therapy, ICPI monotherapy is not considered an evidence-based paradigm; however, in the real world, our data suggest providers periodically take this approach. Technically, the KeyNote-062 trial demonstrated noninferiority for pembrolizumab monotherapy versus chemotherapy and we wondered whether there might be a population of frontline patients who could be considered for ICPI-based approaches or future trials. We were able to evaluate a small cohort of first-line patients who received single-agent ICPI in comparison with patients receiving standard chemotherapy (Fig. 5). While this cohort is underpowered to draw precise conclusions, the directionality and magnitude of the point estimates for comparative drug effectiveness in TMB-stratified patients is consistent with the second-line and sequential cohorts (Figs. 2–4) and subgroup analysis of second-line phase III RCT (ref. 14; Fig. 6). Our data hint that a frontline trial of ICPI versus chemotherapy selected for TMB ≥ 10 might be positive for a first-line chemotherapy-sparing regimen; however, the limited sample size (*n* = 7 ICPI, 59 chemotherapy) must be considered.

We took multiple steps to try and address the limitations of our dataset. Treatment assignments were at the discretion of the clinician, and while biases were carefully considered, known imbalances adjusted, and interpreted in concert with interpatient analyses and compared with existing RCT subgroup analyses, unknown confounders may remain. Although TTNT is a validated real-world data (RWD) measure we recognize that it is possible TTNT may overestimate PFS as there may be a clinical lag time from the time of imaging-based progression to actual initiation of the next treatment. We did not have direct measures of total disease burden at times of treatment. While correlated factors like ECOG were adjusted, ECOG is an imperfect proxy for patient frailty and disease extent which are not fully captured in our dataset. We recognize that TMB calculation can vary considerably by panel size, gene content and bioinformatic filtering (15) and the use of the only FDA-approved TMB companion diagnostic and threshold is a strength of our approach.

## Conclusions

TMB ≥ 10 robustly identifies patients with metastatic gastroesophageal who have more favorable outcomes on first-line single-agent ICPI compared with chemotherapy in patient populations and treatment settings more diverse than registrational clinical trials. While first-line data are limited, the effects seen are not inconsistent with second-line observations. Given the high unmet need in gastroesophageal cancers, consideration for biomarker-enriched prospective 2 L trials incorporating TMB assessment into eligibility is warranted, and may be considered for frontline designs as well.

## Supplementary Material

Supplemental Table S1 Patient demographics in the second line patient cohort.

Supplemental Table S2 Patient demographics in Sequential Cohort

Supplemental Table S3 Patient demographics in 1st Line Cohort

Supplemental Table S4 Summary of TMB ranges per cohort and MSI. The median and interquartile range of TMB is shown per group within the cohorts, grouped by MSI status.

Supplemental Table S5 Summary of TMB ranges per cohort and MSI (TMB10+ only). The median and interquartile range of TMB is shown per group within the cohorts, grouped by MSI status.

Supplemental Table S6 Summary of TMB ranges per cohort and PD-L1. The median and interquartile range of TMB is shown per group within the cohorts, grouped by PD-L1 status.

Supplemental Table S7 Restricted Mean Survival Time Difference by TMB Status in the 2L and 1L Comparative Effectiveness Cohorts. Conditional average treatment effects are defined in each TMB subgroup as the difference in expected survival time up to 3 years when treated with ICPI vs chemo. For instance, within the 1st 3 years after 2nd line treatment initiation, patients with TMB10+ would be expected to have a mean of 15.4 more months until treatment switching on ICPI compared to chemo. Bootstrap 95% confidence intervals (q025, q975) were estimated with 5000 resampling replicates.

Supplemental Table S8 TMB and PD-L1 status and strata in the 2L and sequential cohorts.

Figure S1 Propensity Weighting and Balance Adjustment: TMB. The pre- and post-adjustment balance for (A) 2nd line cohort with TMB < 10, (B) 2nd line cohort with TMB 10 or greater, (C) 1st line cohort with TMB < 10, (D) 1st line cohort with TMB 10 or greater.

Figure S2 2nd line treatment-TMB interaction models from Figure 2. The (A) TTNT and (B) OS interaction models are shown for for propensity adjusted analyses in Figure 2.

Figure S3 Unadjusted 2nd line treatment-TMB interaction models from Supplemental Figure 2. The (A) TTNT and (B) OS interaction models are shown for for propensity adjusted analyses in Supplemental Figure 2.

Figure S4 Unadjusted for known treatment assignment imbalances, patients receiving 2nd line ICPI vs. chemotherapy have more favorable outcomes when TMB ≥ 10 but not TMB < 10. Kaplan-Meier curves are unadjusted for imbalances. TTNT is shown by drug class for (A) TMB < 10, and (B) TMB ≥ 10. OS is shown by drug class for (C) TMB < 10, and (D) TMB ≥ 10. X-axis is truncated at 36 months. Overall survival estimates are left truncated (see Methods) with at-risk tables adjusted accordingly. Interaction models can be found in Supplemental Figure 8.

Figure S5 Sequential TTNT treatment-TMB interaction model from Figure 3. The treatment interaction model from Figure 3C is shown numerically.

Figure S6 Unadjusted 1st line treatment-TMB interaction models from Supplemental Figure 3. The (A) TTNT and (B) OS interaction models are shown for for propensity adjusted analyses in Supplemental Figure 3.

Figure S7 Discriminatory Power Comparison of MSI and TMB. The concordance indexes from univariable Cox PH models containing NGS-based MSI assessment, TMB, or the two in a combined multivariable Cox PH model is shown with (A) TTNT and (B) OS. Error bars indicate standard error.

Figure S8 TMB ≥ 10 treatment predictive associations is not approximated by PD-L1 CPS ≥ 10. Cox PH models containing treatment interaction terms for both TMB ≥ 10 and PD-L1 CPS ≥ 10 are shown numerically (A) 2nd line cohort TTNT, (B) 2nd line cohort OS, and (C) sequential cohort TTNT.

Figure S9 1st line treatment-TMB interaction models from Figure 5. The (A) TTNT and (B) OS interaction models are shown for for propensity adjusted analyses in Figure 5.

Figure S10 Unadjusted for known treatment assignment imbalances, patients receiving 1st line ICPI vs. chemotherapy have more favorable outcomes when TMB ≥ 10 but not TMB < 10. Kaplan-Meier curves are unadjusted for imbalances (propensity weights applied). TTNT is shown by drug class for (A) TMB < 10, and (B) TMB ≥ 10. OS is shown by drug class for (C) TMB < 10, and (D) TMB ≥ 10. X-axis is truncated at 36 months. Overall survival estimates are left truncated (see Methods) with at-risk tables adjusted accordingly. Interaction models can be found in Supplemental Figure 9.
