# Supplementary material for: Real-world Validation of TMB and Microsatellite Instability as Predictive Biomarkers of Immune Checkpoint Inhibitor Effectiveness in Advanced Gastroesophageal Cancer
Source: Cancer Res Commun. 2022 Sep 21;2(9):1037–48. doi: 10.1158/2767-9764.CRC-22-0161 (PMC10010289; doi:10.1158/2767-9764.CRC-22-0161)
Supplement: Supplemental Table S1 — Patient demographics in the second line patient cohort. [file crc-22-0161-s01.pptx]

## Slide 1
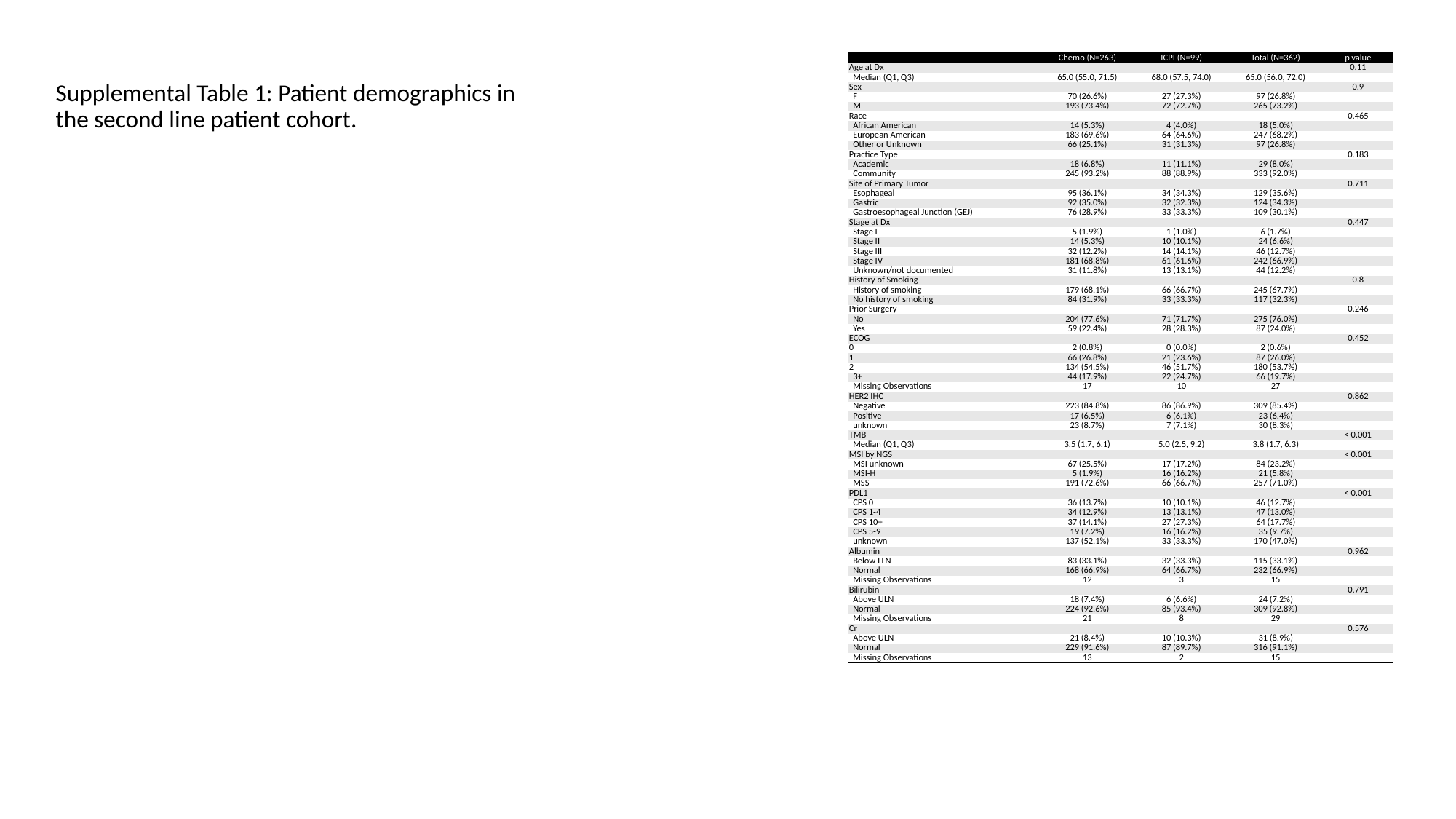

| | Chemo (N=263) | ICPI (N=99) | Total (N=362) | p value |
| --- | --- | --- | --- | --- |
| Age at Dx | | | | 0.11 |
| Median (Q1, Q3) | 65.0 (55.0, 71.5) | 68.0 (57.5, 74.0) | 65.0 (56.0, 72.0) | |
| Sex | | | | 0.9 |
| F | 70 (26.6%) | 27 (27.3%) | 97 (26.8%) | |
| M | 193 (73.4%) | 72 (72.7%) | 265 (73.2%) | |
| Race | | | | 0.465 |
| African American | 14 (5.3%) | 4 (4.0%) | 18 (5.0%) | |
| European American | 183 (69.6%) | 64 (64.6%) | 247 (68.2%) | |
| Other or Unknown | 66 (25.1%) | 31 (31.3%) | 97 (26.8%) | |
| Practice Type | | | | 0.183 |
| Academic | 18 (6.8%) | 11 (11.1%) | 29 (8.0%) | |
| Community | 245 (93.2%) | 88 (88.9%) | 333 (92.0%) | |
| Site of Primary Tumor | | | | 0.711 |
| Esophageal | 95 (36.1%) | 34 (34.3%) | 129 (35.6%) | |
| Gastric | 92 (35.0%) | 32 (32.3%) | 124 (34.3%) | |
| Gastroesophageal Junction (GEJ) | 76 (28.9%) | 33 (33.3%) | 109 (30.1%) | |
| Stage at Dx | | | | 0.447 |
| Stage I | 5 (1.9%) | 1 (1.0%) | 6 (1.7%) | |
| Stage II | 14 (5.3%) | 10 (10.1%) | 24 (6.6%) | |
| Stage III | 32 (12.2%) | 14 (14.1%) | 46 (12.7%) | |
| Stage IV | 181 (68.8%) | 61 (61.6%) | 242 (66.9%) | |
| Unknown/not documented | 31 (11.8%) | 13 (13.1%) | 44 (12.2%) | |
| History of Smoking | | | | 0.8 |
| History of smoking | 179 (68.1%) | 66 (66.7%) | 245 (67.7%) | |
| No history of smoking | 84 (31.9%) | 33 (33.3%) | 117 (32.3%) | |
| Prior Surgery | | | | 0.246 |
| No | 204 (77.6%) | 71 (71.7%) | 275 (76.0%) | |
| Yes | 59 (22.4%) | 28 (28.3%) | 87 (24.0%) | |
| ECOG | | | | 0.452 |
| 0 | 2 (0.8%) | 0 (0.0%) | 2 (0.6%) | |
| 1 | 66 (26.8%) | 21 (23.6%) | 87 (26.0%) | |
| 2 | 134 (54.5%) | 46 (51.7%) | 180 (53.7%) | |
| 3+ | 44 (17.9%) | 22 (24.7%) | 66 (19.7%) | |
| Missing Observations | 17 | 10 | 27 | |
| HER2 IHC | | | | 0.862 |
| Negative | 223 (84.8%) | 86 (86.9%) | 309 (85.4%) | |
| Positive | 17 (6.5%) | 6 (6.1%) | 23 (6.4%) | |
| unknown | 23 (8.7%) | 7 (7.1%) | 30 (8.3%) | |
| TMB | | | | < 0.001 |
| Median (Q1, Q3) | 3.5 (1.7, 6.1) | 5.0 (2.5, 9.2) | 3.8 (1.7, 6.3) | |
| MSI by NGS | | | | < 0.001 |
| MSI unknown | 67 (25.5%) | 17 (17.2%) | 84 (23.2%) | |
| MSI-H | 5 (1.9%) | 16 (16.2%) | 21 (5.8%) | |
| MSS | 191 (72.6%) | 66 (66.7%) | 257 (71.0%) | |
| PDL1 | | | | < 0.001 |
| CPS 0 | 36 (13.7%) | 10 (10.1%) | 46 (12.7%) | |
| CPS 1-4 | 34 (12.9%) | 13 (13.1%) | 47 (13.0%) | |
| CPS 10+ | 37 (14.1%) | 27 (27.3%) | 64 (17.7%) | |
| CPS 5-9 | 19 (7.2%) | 16 (16.2%) | 35 (9.7%) | |
| unknown | 137 (52.1%) | 33 (33.3%) | 170 (47.0%) | |
| Albumin | | | | 0.962 |
| Below LLN | 83 (33.1%) | 32 (33.3%) | 115 (33.1%) | |
| Normal | 168 (66.9%) | 64 (66.7%) | 232 (66.9%) | |
| Missing Observations | 12 | 3 | 15 | |
| Bilirubin | | | | 0.791 |
| Above ULN | 18 (7.4%) | 6 (6.6%) | 24 (7.2%) | |
| Normal | 224 (92.6%) | 85 (93.4%) | 309 (92.8%) | |
| Missing Observations | 21 | 8 | 29 | |
| Cr | | | | 0.576 |
| Above ULN | 21 (8.4%) | 10 (10.3%) | 31 (8.9%) | |
| Normal | 229 (91.6%) | 87 (89.7%) | 316 (91.1%) | |
| Missing Observations | 13 | 2 | 15 | |
# Supplemental Table 1: Patient demographics in the second line patient cohort.
