# Supplementary material for: Real-world Validation of TMB and Microsatellite Instability as Predictive Biomarkers of Immune Checkpoint Inhibitor Effectiveness in Advanced Gastroesophageal Cancer
Source: Cancer Res Commun. 2022 Sep 21;2(9):1037–48. doi: 10.1158/2767-9764.CRC-22-0161 (PMC10010289; doi:10.1158/2767-9764.CRC-22-0161)
Supplement: Supplemental Table S2 — Patient demographics in Sequential Cohort [file crc-22-0161-s02.pptx]

## Slide 1
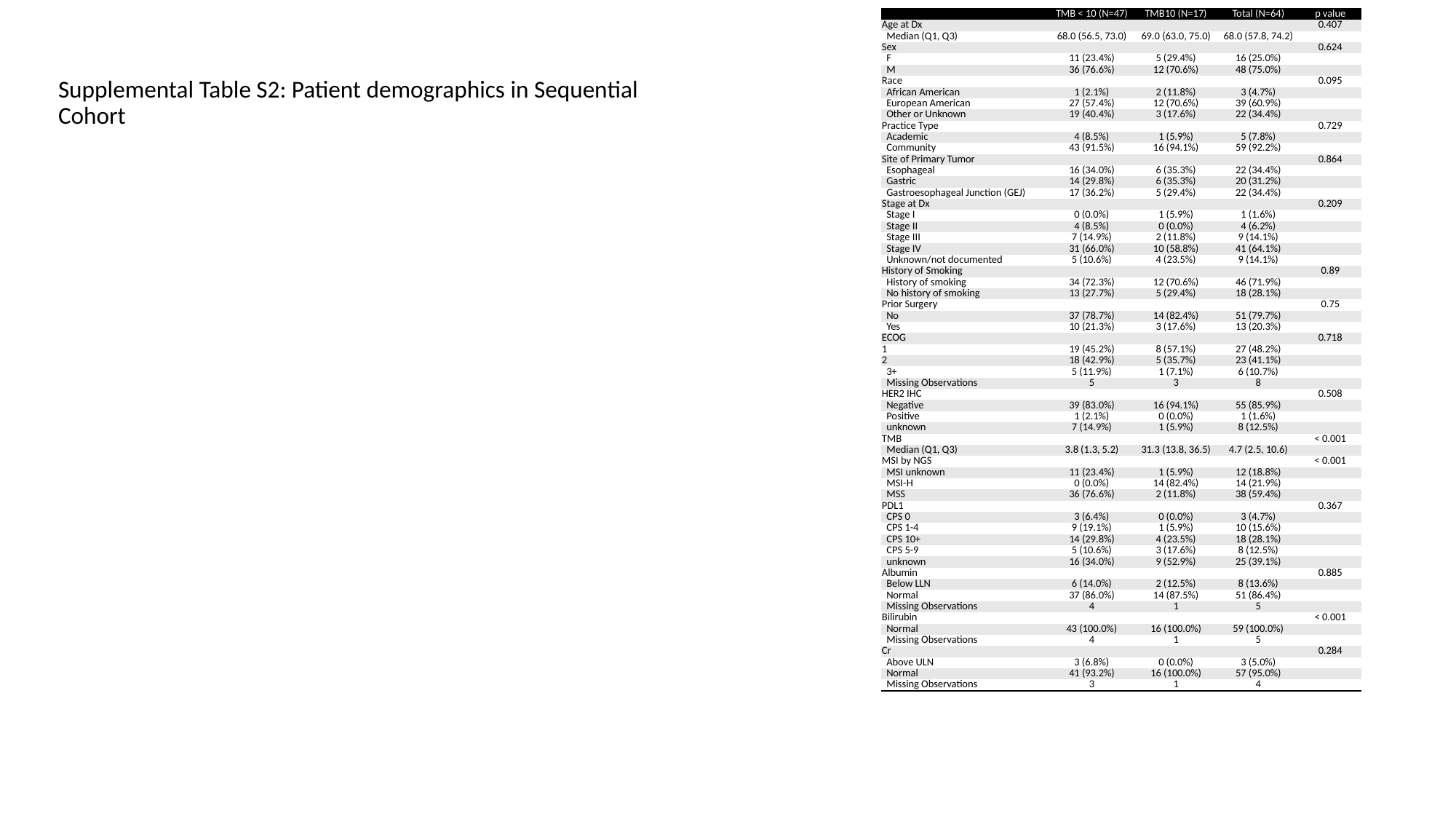

| | TMB < 10 (N=47) | TMB10 (N=17) | Total (N=64) | p value |
| --- | --- | --- | --- | --- |
| Age at Dx | | | | 0.407 |
| Median (Q1, Q3) | 68.0 (56.5, 73.0) | 69.0 (63.0, 75.0) | 68.0 (57.8, 74.2) | |
| Sex | | | | 0.624 |
| F | 11 (23.4%) | 5 (29.4%) | 16 (25.0%) | |
| M | 36 (76.6%) | 12 (70.6%) | 48 (75.0%) | |
| Race | | | | 0.095 |
| African American | 1 (2.1%) | 2 (11.8%) | 3 (4.7%) | |
| European American | 27 (57.4%) | 12 (70.6%) | 39 (60.9%) | |
| Other or Unknown | 19 (40.4%) | 3 (17.6%) | 22 (34.4%) | |
| Practice Type | | | | 0.729 |
| Academic | 4 (8.5%) | 1 (5.9%) | 5 (7.8%) | |
| Community | 43 (91.5%) | 16 (94.1%) | 59 (92.2%) | |
| Site of Primary Tumor | | | | 0.864 |
| Esophageal | 16 (34.0%) | 6 (35.3%) | 22 (34.4%) | |
| Gastric | 14 (29.8%) | 6 (35.3%) | 20 (31.2%) | |
| Gastroesophageal Junction (GEJ) | 17 (36.2%) | 5 (29.4%) | 22 (34.4%) | |
| Stage at Dx | | | | 0.209 |
| Stage I | 0 (0.0%) | 1 (5.9%) | 1 (1.6%) | |
| Stage II | 4 (8.5%) | 0 (0.0%) | 4 (6.2%) | |
| Stage III | 7 (14.9%) | 2 (11.8%) | 9 (14.1%) | |
| Stage IV | 31 (66.0%) | 10 (58.8%) | 41 (64.1%) | |
| Unknown/not documented | 5 (10.6%) | 4 (23.5%) | 9 (14.1%) | |
| History of Smoking | | | | 0.89 |
| History of smoking | 34 (72.3%) | 12 (70.6%) | 46 (71.9%) | |
| No history of smoking | 13 (27.7%) | 5 (29.4%) | 18 (28.1%) | |
| Prior Surgery | | | | 0.75 |
| No | 37 (78.7%) | 14 (82.4%) | 51 (79.7%) | |
| Yes | 10 (21.3%) | 3 (17.6%) | 13 (20.3%) | |
| ECOG | | | | 0.718 |
| 1 | 19 (45.2%) | 8 (57.1%) | 27 (48.2%) | |
| 2 | 18 (42.9%) | 5 (35.7%) | 23 (41.1%) | |
| 3+ | 5 (11.9%) | 1 (7.1%) | 6 (10.7%) | |
| Missing Observations | 5 | 3 | 8 | |
| HER2 IHC | | | | 0.508 |
| Negative | 39 (83.0%) | 16 (94.1%) | 55 (85.9%) | |
| Positive | 1 (2.1%) | 0 (0.0%) | 1 (1.6%) | |
| unknown | 7 (14.9%) | 1 (5.9%) | 8 (12.5%) | |
| TMB | | | | < 0.001 |
| Median (Q1, Q3) | 3.8 (1.3, 5.2) | 31.3 (13.8, 36.5) | 4.7 (2.5, 10.6) | |
| MSI by NGS | | | | < 0.001 |
| MSI unknown | 11 (23.4%) | 1 (5.9%) | 12 (18.8%) | |
| MSI-H | 0 (0.0%) | 14 (82.4%) | 14 (21.9%) | |
| MSS | 36 (76.6%) | 2 (11.8%) | 38 (59.4%) | |
| PDL1 | | | | 0.367 |
| CPS 0 | 3 (6.4%) | 0 (0.0%) | 3 (4.7%) | |
| CPS 1-4 | 9 (19.1%) | 1 (5.9%) | 10 (15.6%) | |
| CPS 10+ | 14 (29.8%) | 4 (23.5%) | 18 (28.1%) | |
| CPS 5-9 | 5 (10.6%) | 3 (17.6%) | 8 (12.5%) | |
| unknown | 16 (34.0%) | 9 (52.9%) | 25 (39.1%) | |
| Albumin | | | | 0.885 |
| Below LLN | 6 (14.0%) | 2 (12.5%) | 8 (13.6%) | |
| Normal | 37 (86.0%) | 14 (87.5%) | 51 (86.4%) | |
| Missing Observations | 4 | 1 | 5 | |
| Bilirubin | | | | < 0.001 |
| Normal | 43 (100.0%) | 16 (100.0%) | 59 (100.0%) | |
| Missing Observations | 4 | 1 | 5 | |
| Cr | | | | 0.284 |
| Above ULN | 3 (6.8%) | 0 (0.0%) | 3 (5.0%) | |
| Normal | 41 (93.2%) | 16 (100.0%) | 57 (95.0%) | |
| Missing Observations | 3 | 1 | 4 | |
# Supplemental Table S2: Patient demographics in Sequential Cohort
