# Supplementary material for: Real-world Validation of TMB and Microsatellite Instability as Predictive Biomarkers of Immune Checkpoint Inhibitor Effectiveness in Advanced Gastroesophageal Cancer
Source: Cancer Res Commun. 2022 Sep 21;2(9):1037–48. doi: 10.1158/2767-9764.CRC-22-0161 (PMC10010289; doi:10.1158/2767-9764.CRC-22-0161)
Supplement: Supplemental Table S3 — Patient demographics in 1st Line Cohort [file crc-22-0161-s03.pptx]

## Slide 1
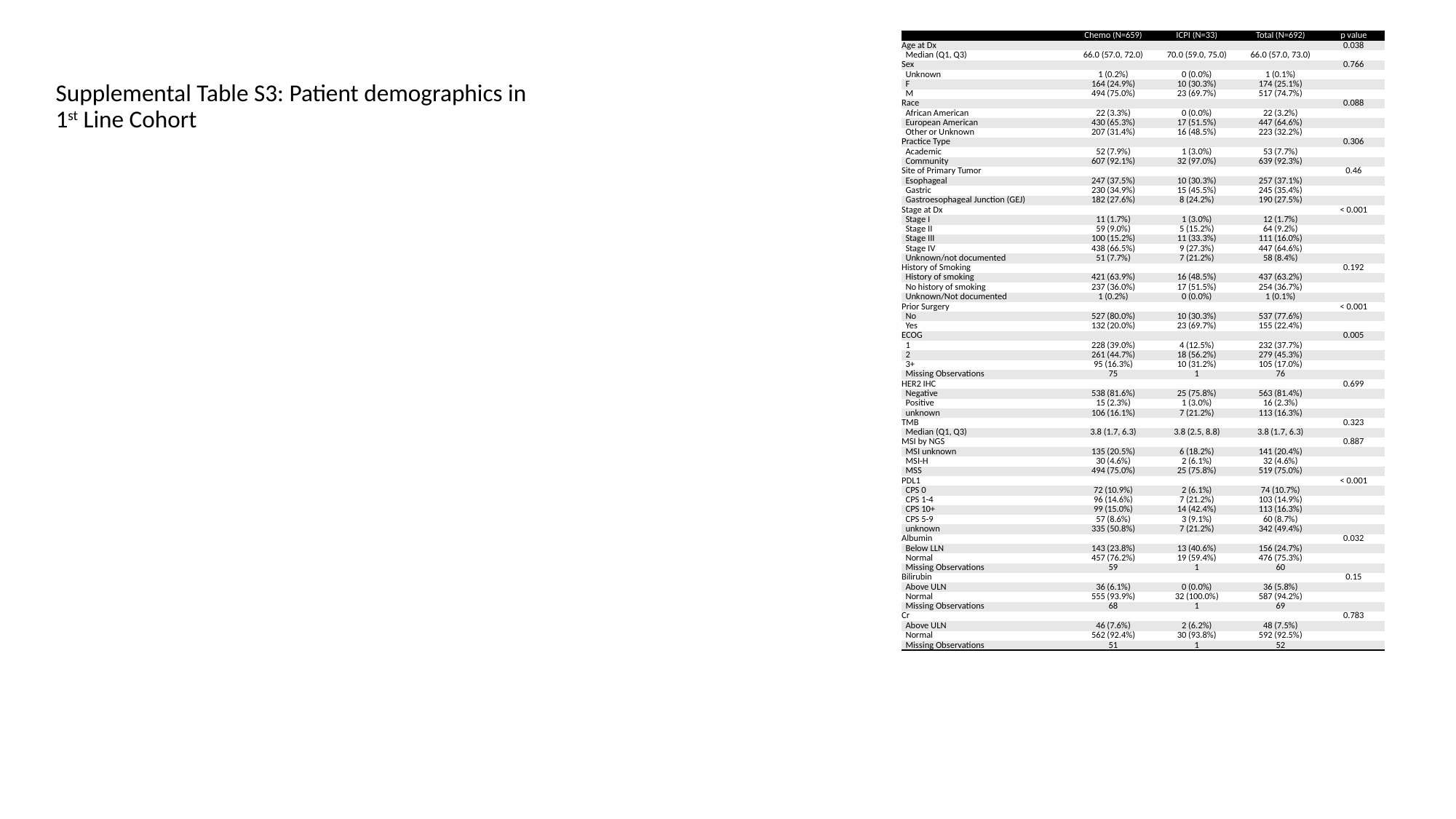

| | Chemo (N=659) | ICPI (N=33) | Total (N=692) | p value |
| --- | --- | --- | --- | --- |
| Age at Dx | | | | 0.038 |
| Median (Q1, Q3) | 66.0 (57.0, 72.0) | 70.0 (59.0, 75.0) | 66.0 (57.0, 73.0) | |
| Sex | | | | 0.766 |
| Unknown | 1 (0.2%) | 0 (0.0%) | 1 (0.1%) | |
| F | 164 (24.9%) | 10 (30.3%) | 174 (25.1%) | |
| M | 494 (75.0%) | 23 (69.7%) | 517 (74.7%) | |
| Race | | | | 0.088 |
| African American | 22 (3.3%) | 0 (0.0%) | 22 (3.2%) | |
| European American | 430 (65.3%) | 17 (51.5%) | 447 (64.6%) | |
| Other or Unknown | 207 (31.4%) | 16 (48.5%) | 223 (32.2%) | |
| Practice Type | | | | 0.306 |
| Academic | 52 (7.9%) | 1 (3.0%) | 53 (7.7%) | |
| Community | 607 (92.1%) | 32 (97.0%) | 639 (92.3%) | |
| Site of Primary Tumor | | | | 0.46 |
| Esophageal | 247 (37.5%) | 10 (30.3%) | 257 (37.1%) | |
| Gastric | 230 (34.9%) | 15 (45.5%) | 245 (35.4%) | |
| Gastroesophageal Junction (GEJ) | 182 (27.6%) | 8 (24.2%) | 190 (27.5%) | |
| Stage at Dx | | | | < 0.001 |
| Stage I | 11 (1.7%) | 1 (3.0%) | 12 (1.7%) | |
| Stage II | 59 (9.0%) | 5 (15.2%) | 64 (9.2%) | |
| Stage III | 100 (15.2%) | 11 (33.3%) | 111 (16.0%) | |
| Stage IV | 438 (66.5%) | 9 (27.3%) | 447 (64.6%) | |
| Unknown/not documented | 51 (7.7%) | 7 (21.2%) | 58 (8.4%) | |
| History of Smoking | | | | 0.192 |
| History of smoking | 421 (63.9%) | 16 (48.5%) | 437 (63.2%) | |
| No history of smoking | 237 (36.0%) | 17 (51.5%) | 254 (36.7%) | |
| Unknown/Not documented | 1 (0.2%) | 0 (0.0%) | 1 (0.1%) | |
| Prior Surgery | | | | < 0.001 |
| No | 527 (80.0%) | 10 (30.3%) | 537 (77.6%) | |
| Yes | 132 (20.0%) | 23 (69.7%) | 155 (22.4%) | |
| ECOG | | | | 0.005 |
| 1 | 228 (39.0%) | 4 (12.5%) | 232 (37.7%) | |
| 2 | 261 (44.7%) | 18 (56.2%) | 279 (45.3%) | |
| 3+ | 95 (16.3%) | 10 (31.2%) | 105 (17.0%) | |
| Missing Observations | 75 | 1 | 76 | |
| HER2 IHC | | | | 0.699 |
| Negative | 538 (81.6%) | 25 (75.8%) | 563 (81.4%) | |
| Positive | 15 (2.3%) | 1 (3.0%) | 16 (2.3%) | |
| unknown | 106 (16.1%) | 7 (21.2%) | 113 (16.3%) | |
| TMB | | | | 0.323 |
| Median (Q1, Q3) | 3.8 (1.7, 6.3) | 3.8 (2.5, 8.8) | 3.8 (1.7, 6.3) | |
| MSI by NGS | | | | 0.887 |
| MSI unknown | 135 (20.5%) | 6 (18.2%) | 141 (20.4%) | |
| MSI-H | 30 (4.6%) | 2 (6.1%) | 32 (4.6%) | |
| MSS | 494 (75.0%) | 25 (75.8%) | 519 (75.0%) | |
| PDL1 | | | | < 0.001 |
| CPS 0 | 72 (10.9%) | 2 (6.1%) | 74 (10.7%) | |
| CPS 1-4 | 96 (14.6%) | 7 (21.2%) | 103 (14.9%) | |
| CPS 10+ | 99 (15.0%) | 14 (42.4%) | 113 (16.3%) | |
| CPS 5-9 | 57 (8.6%) | 3 (9.1%) | 60 (8.7%) | |
| unknown | 335 (50.8%) | 7 (21.2%) | 342 (49.4%) | |
| Albumin | | | | 0.032 |
| Below LLN | 143 (23.8%) | 13 (40.6%) | 156 (24.7%) | |
| Normal | 457 (76.2%) | 19 (59.4%) | 476 (75.3%) | |
| Missing Observations | 59 | 1 | 60 | |
| Bilirubin | | | | 0.15 |
| Above ULN | 36 (6.1%) | 0 (0.0%) | 36 (5.8%) | |
| Normal | 555 (93.9%) | 32 (100.0%) | 587 (94.2%) | |
| Missing Observations | 68 | 1 | 69 | |
| Cr | | | | 0.783 |
| Above ULN | 46 (7.6%) | 2 (6.2%) | 48 (7.5%) | |
| Normal | 562 (92.4%) | 30 (93.8%) | 592 (92.5%) | |
| Missing Observations | 51 | 1 | 52 | |
# Supplemental Table S3: Patient demographics in 1st Line Cohort
