# Supplementary material for: Real-world Validation of TMB and Microsatellite Instability as Predictive Biomarkers of Immune Checkpoint Inhibitor Effectiveness in Advanced Gastroesophageal Cancer
Source: Cancer Res Commun. 2022 Sep 21;2(9):1037–48. doi: 10.1158/2767-9764.CRC-22-0161 (PMC10010289; doi:10.1158/2767-9764.CRC-22-0161)
Supplement: Supplemental Table S4 — Summary of TMB ranges per cohort and MSI. The median and interquartile range of TMB is shown per group within the cohorts, grouped by MSI status. [file crc-22-0161-s04.pptx]

## Slide 1
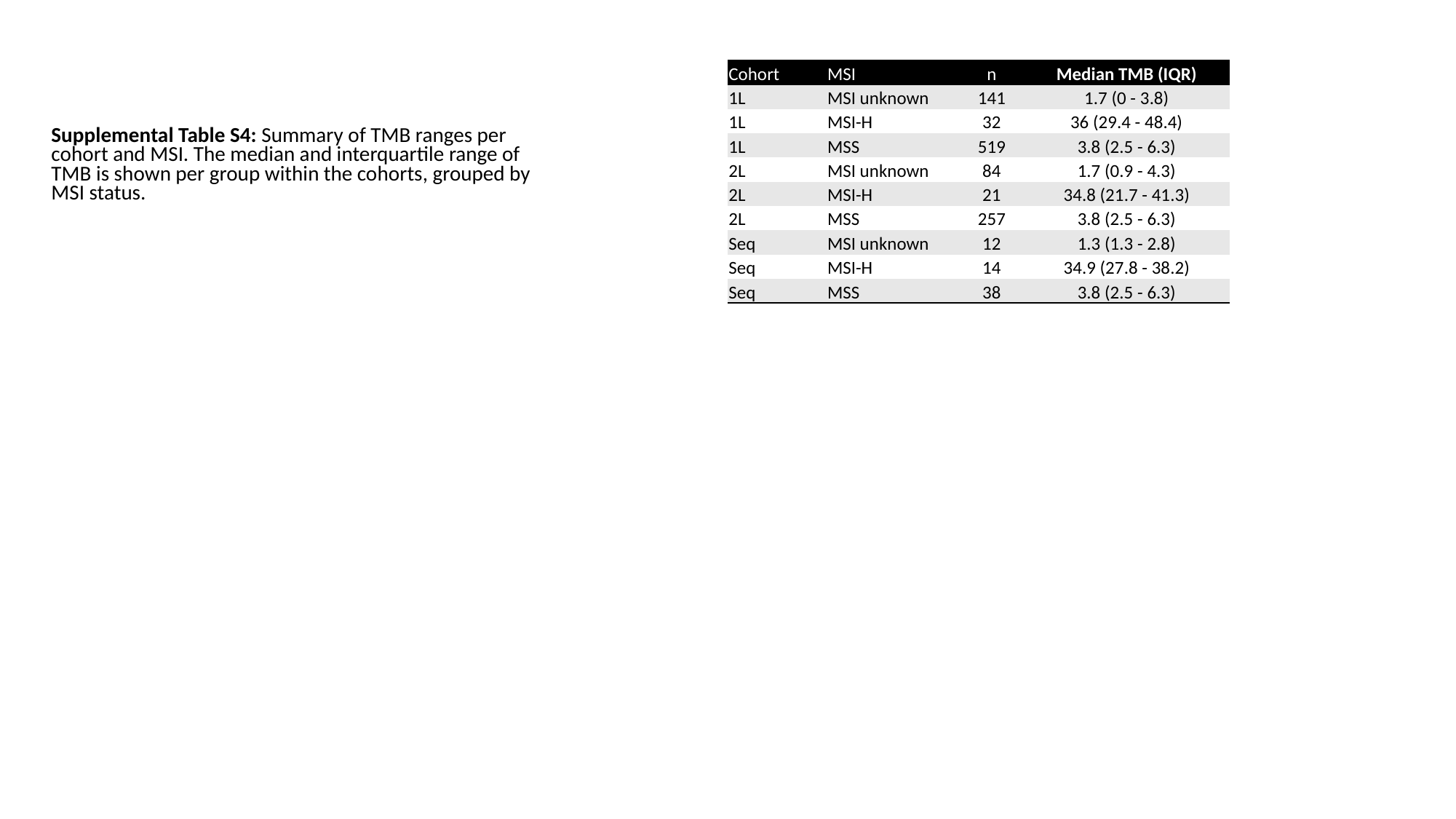

| Cohort | MSI | n | Median TMB (IQR) |
| --- | --- | --- | --- |
| 1L | MSI unknown | 141 | 1.7 (0 - 3.8) |
| 1L | MSI-H | 32 | 36 (29.4 - 48.4) |
| 1L | MSS | 519 | 3.8 (2.5 - 6.3) |
| 2L | MSI unknown | 84 | 1.7 (0.9 - 4.3) |
| 2L | MSI-H | 21 | 34.8 (21.7 - 41.3) |
| 2L | MSS | 257 | 3.8 (2.5 - 6.3) |
| Seq | MSI unknown | 12 | 1.3 (1.3 - 2.8) |
| Seq | MSI-H | 14 | 34.9 (27.8 - 38.2) |
| Seq | MSS | 38 | 3.8 (2.5 - 6.3) |
Supplemental Table S4: Summary of TMB ranges per cohort and MSI. The median and interquartile range of TMB is shown per group within the cohorts, grouped by MSI status.
