# Supplementary material for: Real-world Validation of TMB and Microsatellite Instability as Predictive Biomarkers of Immune Checkpoint Inhibitor Effectiveness in Advanced Gastroesophageal Cancer
Source: Cancer Res Commun. 2022 Sep 21;2(9):1037–48. doi: 10.1158/2767-9764.CRC-22-0161 (PMC10010289; doi:10.1158/2767-9764.CRC-22-0161)
Supplement: Supplemental Table S5 — Summary of TMB ranges per cohort and MSI (TMB10+ only). The median and interquartile range of TMB is shown per group within the cohorts, grouped by MSI status. [file crc-22-0161-s05.pptx]

## Slide 1
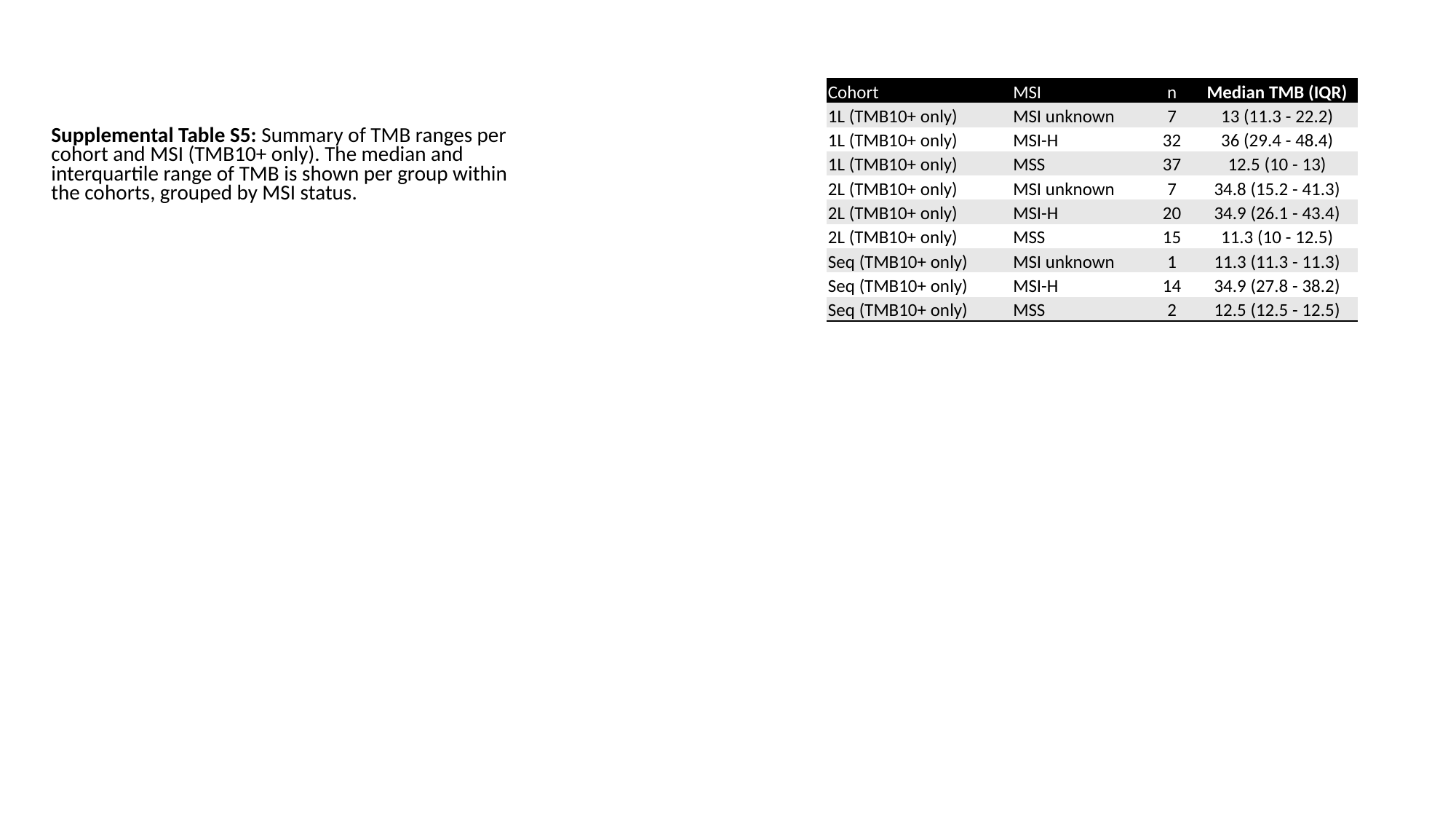

| Cohort | MSI | n | Median TMB (IQR) |
| --- | --- | --- | --- |
| 1L (TMB10+ only) | MSI unknown | 7 | 13 (11.3 - 22.2) |
| 1L (TMB10+ only) | MSI-H | 32 | 36 (29.4 - 48.4) |
| 1L (TMB10+ only) | MSS | 37 | 12.5 (10 - 13) |
| 2L (TMB10+ only) | MSI unknown | 7 | 34.8 (15.2 - 41.3) |
| 2L (TMB10+ only) | MSI-H | 20 | 34.9 (26.1 - 43.4) |
| 2L (TMB10+ only) | MSS | 15 | 11.3 (10 - 12.5) |
| Seq (TMB10+ only) | MSI unknown | 1 | 11.3 (11.3 - 11.3) |
| Seq (TMB10+ only) | MSI-H | 14 | 34.9 (27.8 - 38.2) |
| Seq (TMB10+ only) | MSS | 2 | 12.5 (12.5 - 12.5) |
Supplemental Table S5: Summary of TMB ranges per cohort and MSI (TMB10+ only). The median and interquartile range of TMB is shown per group within the cohorts, grouped by MSI status.
