# Supplementary material for: Real-world Validation of TMB and Microsatellite Instability as Predictive Biomarkers of Immune Checkpoint Inhibitor Effectiveness in Advanced Gastroesophageal Cancer
Source: Cancer Res Commun. 2022 Sep 21;2(9):1037–48. doi: 10.1158/2767-9764.CRC-22-0161 (PMC10010289; doi:10.1158/2767-9764.CRC-22-0161)
Supplement: Supplemental Table S6 — Summary of TMB ranges per cohort and PD-L1. The median and interquartile range of TMB is shown per group within the cohorts, grouped by PD-L1 status. [file crc-22-0161-s06.pptx]

## Slide 1
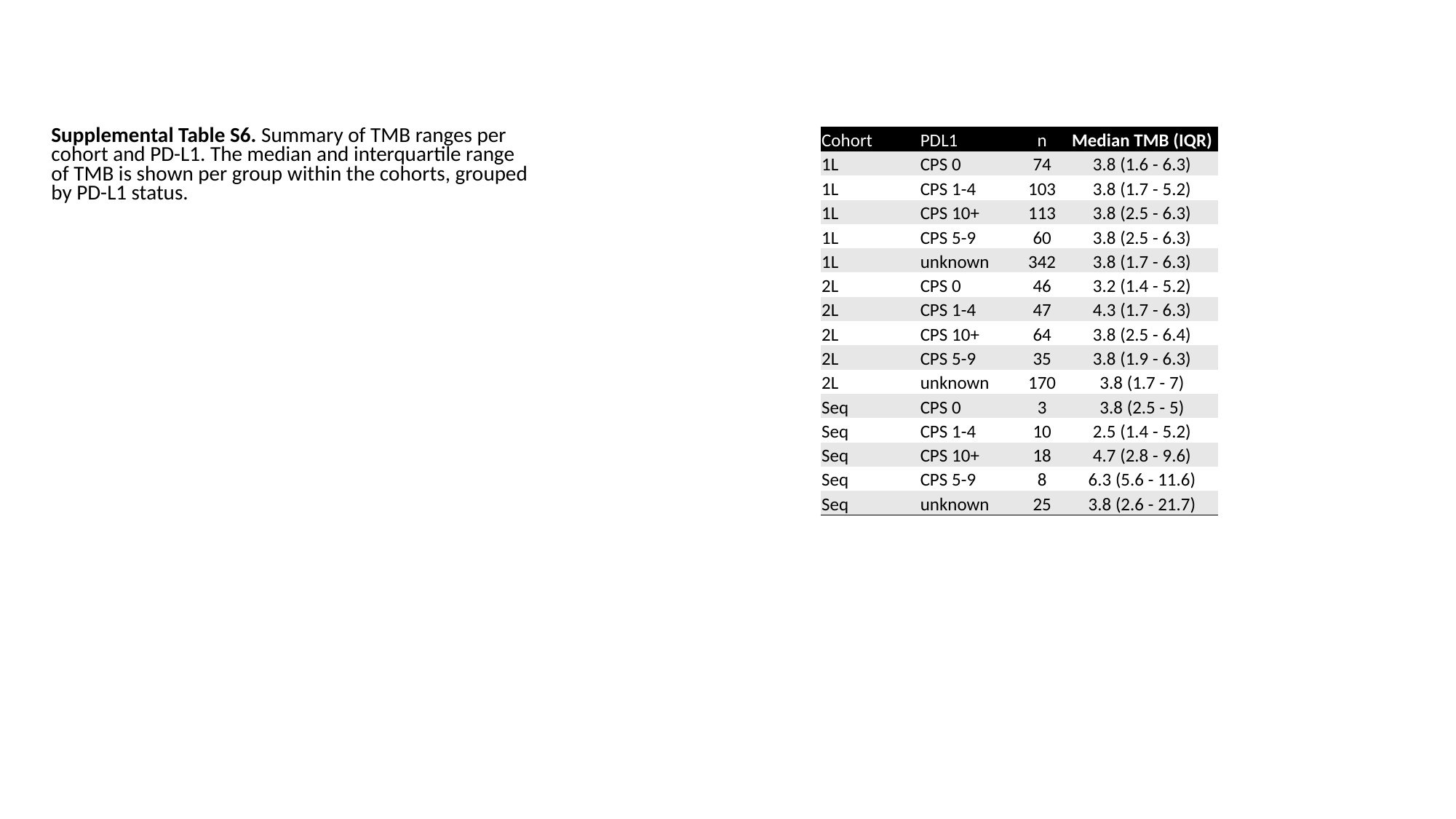

Supplemental Table S6. Summary of TMB ranges per cohort and PD-L1. The median and interquartile range of TMB is shown per group within the cohorts, grouped by PD-L1 status.
| Cohort | PDL1 | n | Median TMB (IQR) |
| --- | --- | --- | --- |
| 1L | CPS 0 | 74 | 3.8 (1.6 - 6.3) |
| 1L | CPS 1-4 | 103 | 3.8 (1.7 - 5.2) |
| 1L | CPS 10+ | 113 | 3.8 (2.5 - 6.3) |
| 1L | CPS 5-9 | 60 | 3.8 (2.5 - 6.3) |
| 1L | unknown | 342 | 3.8 (1.7 - 6.3) |
| 2L | CPS 0 | 46 | 3.2 (1.4 - 5.2) |
| 2L | CPS 1-4 | 47 | 4.3 (1.7 - 6.3) |
| 2L | CPS 10+ | 64 | 3.8 (2.5 - 6.4) |
| 2L | CPS 5-9 | 35 | 3.8 (1.9 - 6.3) |
| 2L | unknown | 170 | 3.8 (1.7 - 7) |
| Seq | CPS 0 | 3 | 3.8 (2.5 - 5) |
| Seq | CPS 1-4 | 10 | 2.5 (1.4 - 5.2) |
| Seq | CPS 10+ | 18 | 4.7 (2.8 - 9.6) |
| Seq | CPS 5-9 | 8 | 6.3 (5.6 - 11.6) |
| Seq | unknown | 25 | 3.8 (2.6 - 21.7) |
