# Supplementary material for: Real-world Validation of TMB and Microsatellite Instability as Predictive Biomarkers of Immune Checkpoint Inhibitor Effectiveness in Advanced Gastroesophageal Cancer
Source: Cancer Res Commun. 2022 Sep 21;2(9):1037–48. doi: 10.1158/2767-9764.CRC-22-0161 (PMC10010289; doi:10.1158/2767-9764.CRC-22-0161)
Supplement: Supplemental Table S7 — Restricted Mean Survival Time Difference by TMB Status in the 2L and 1L Comparative Effectiveness Cohorts. Conditional average treatment effects are defined in each TMB subgroup as the difference in expected survival time up to 3 years when treated with ICPI vs chemo. For instance, within the 1st 3 years after 2nd line treatment initiation, patients with TMB10+ would be expected to have a mean of 15.4 more months until treatment switching on ICPI compared to chemo. Bootstrap 95% confidence intervals (q025, q975) were estimated with 5000 resampling replicates. [file crc-22-0161-s07.pptx]

## Slide 1
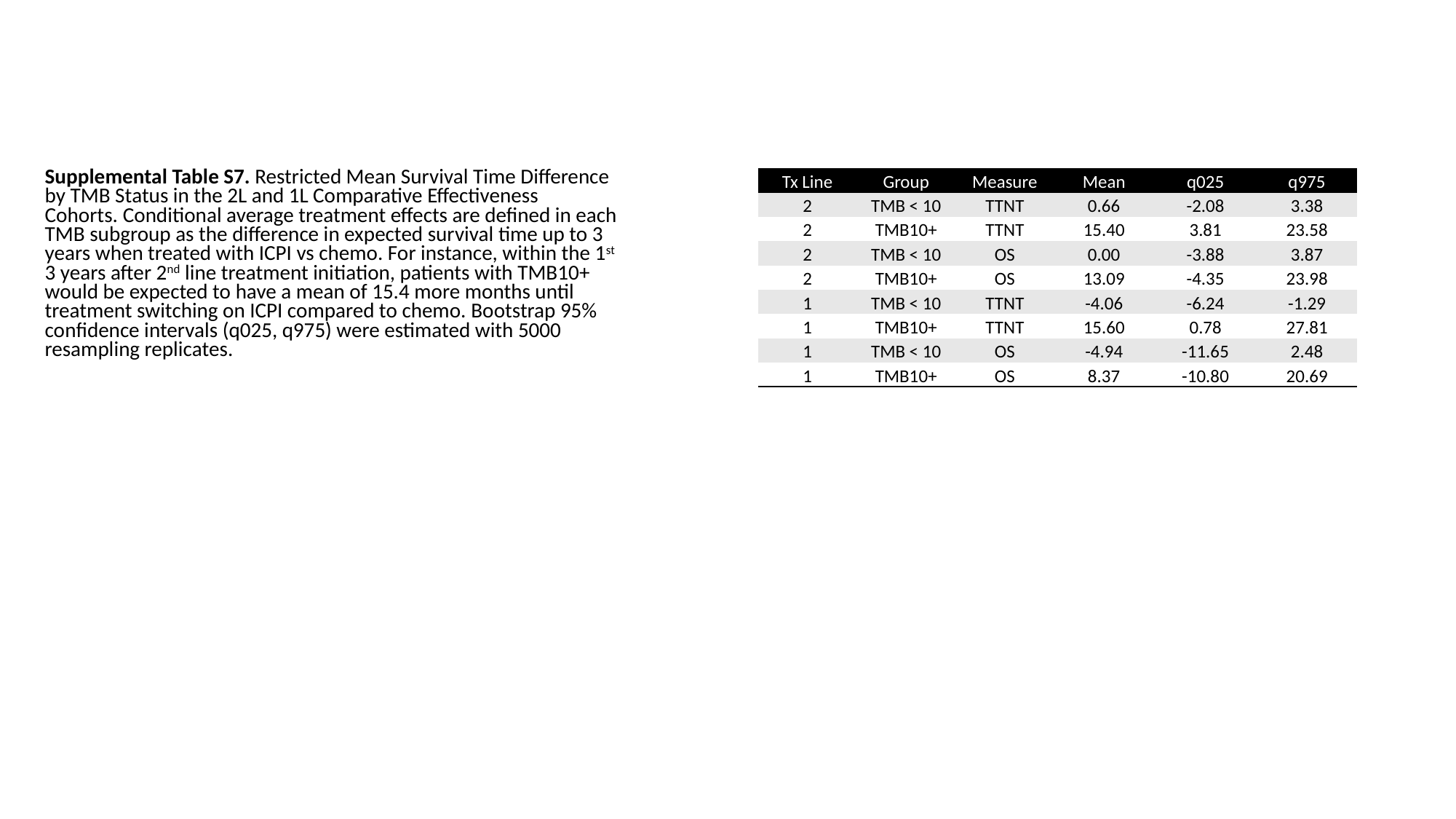

Supplemental Table S7. Restricted Mean Survival Time Difference by TMB Status in the 2L and 1L Comparative Effectiveness Cohorts. Conditional average treatment effects are defined in each TMB subgroup as the difference in expected survival time up to 3 years when treated with ICPI vs chemo. For instance, within the 1st 3 years after 2nd line treatment initiation, patients with TMB10+ would be expected to have a mean of 15.4 more months until treatment switching on ICPI compared to chemo. Bootstrap 95% confidence intervals (q025, q975) were estimated with 5000 resampling replicates.
| Tx Line | Group | Measure | Mean | q025 | q975 |
| --- | --- | --- | --- | --- | --- |
| 2 | TMB < 10 | TTNT | 0.66 | -2.08 | 3.38 |
| 2 | TMB10+ | TTNT | 15.40 | 3.81 | 23.58 |
| 2 | TMB < 10 | OS | 0.00 | -3.88 | 3.87 |
| 2 | TMB10+ | OS | 13.09 | -4.35 | 23.98 |
| 1 | TMB < 10 | TTNT | -4.06 | -6.24 | -1.29 |
| 1 | TMB10+ | TTNT | 15.60 | 0.78 | 27.81 |
| 1 | TMB < 10 | OS | -4.94 | -11.65 | 2.48 |
| 1 | TMB10+ | OS | 8.37 | -10.80 | 20.69 |
