# Supplementary material for: Real-world Validation of TMB and Microsatellite Instability as Predictive Biomarkers of Immune Checkpoint Inhibitor Effectiveness in Advanced Gastroesophageal Cancer
Source: Cancer Res Commun. 2022 Sep 21;2(9):1037–48. doi: 10.1158/2767-9764.CRC-22-0161 (PMC10010289; doi:10.1158/2767-9764.CRC-22-0161)
Supplement: Supplemental Table S8 — TMB and PD-L1 status and strata in the 2L and sequential cohorts. [file crc-22-0161-s08.pptx]

## Slide 1
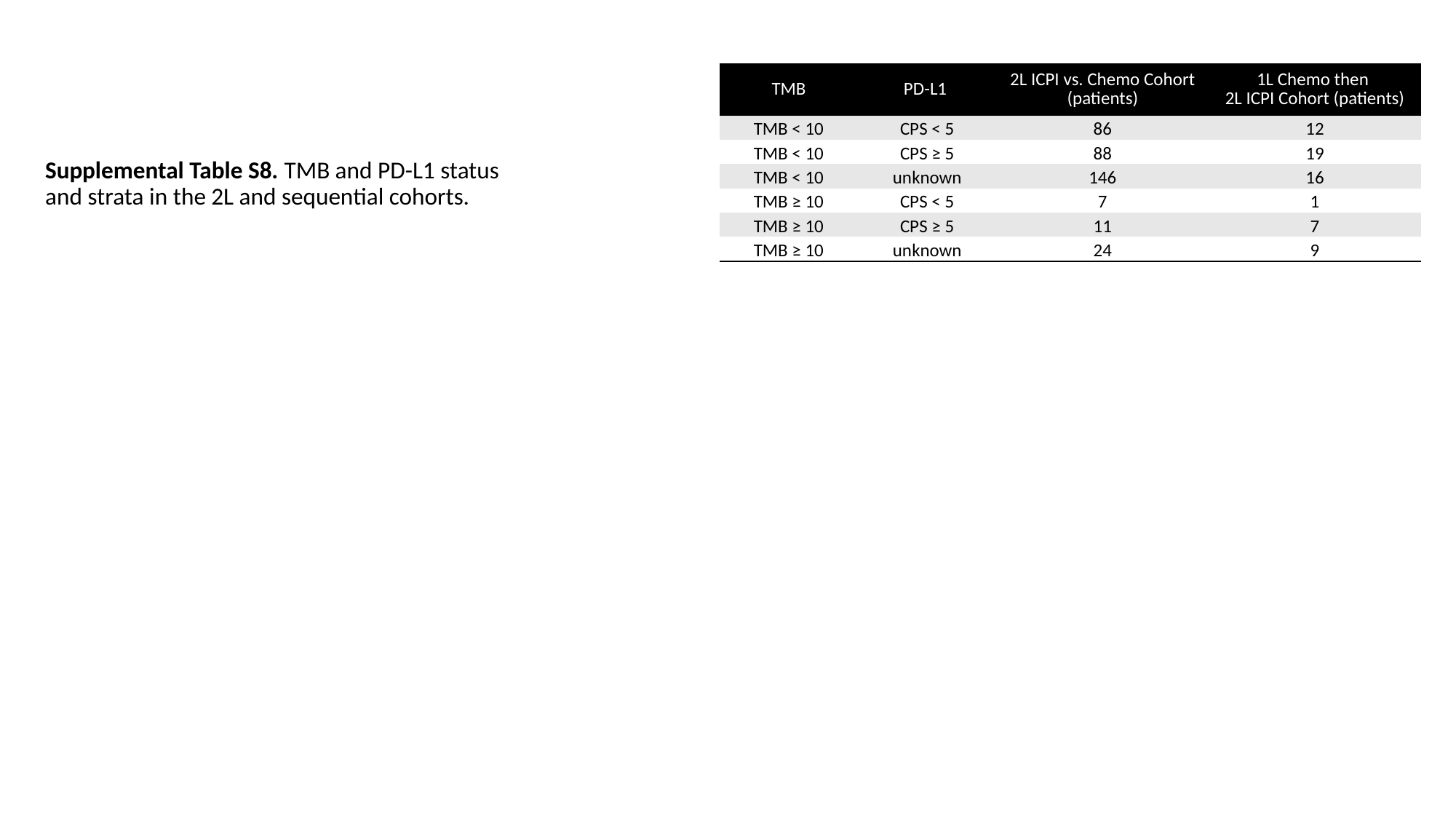

| TMB | PD-L1 | 2L ICPI vs. Chemo Cohort (patients) | 1L Chemo then 2L ICPI Cohort (patients) |
| --- | --- | --- | --- |
| TMB < 10 | CPS < 5 | 86 | 12 |
| TMB < 10 | CPS ≥ 5 | 88 | 19 |
| TMB < 10 | unknown | 146 | 16 |
| TMB ≥ 10 | CPS < 5 | 7 | 1 |
| TMB ≥ 10 | CPS ≥ 5 | 11 | 7 |
| TMB ≥ 10 | unknown | 24 | 9 |
# Supplemental Table S8. TMB and PD-L1 status and strata in the 2L and sequential cohorts.
