# Supplementary material for: Real-world Validation of TMB and Microsatellite Instability as Predictive Biomarkers of Immune Checkpoint Inhibitor Effectiveness in Advanced Gastroesophageal Cancer
Source: Cancer Res Commun. 2022 Sep 21;2(9):1037–48. doi: 10.1158/2767-9764.CRC-22-0161 (PMC10010289; doi:10.1158/2767-9764.CRC-22-0161)
Supplement: Figure S1 — Propensity Weighting and Balance Adjustment: TMB. The pre- and post-adjustment balance for (A) 2nd line cohort with TMB < 10, (B) 2nd line cohort with TMB 10 or greater, (C) 1st line cohort with TMB < 10, (D) 1st line cohort with TMB 10 or greater. [file crc-22-0161-s09.pptx]

## Slide 1
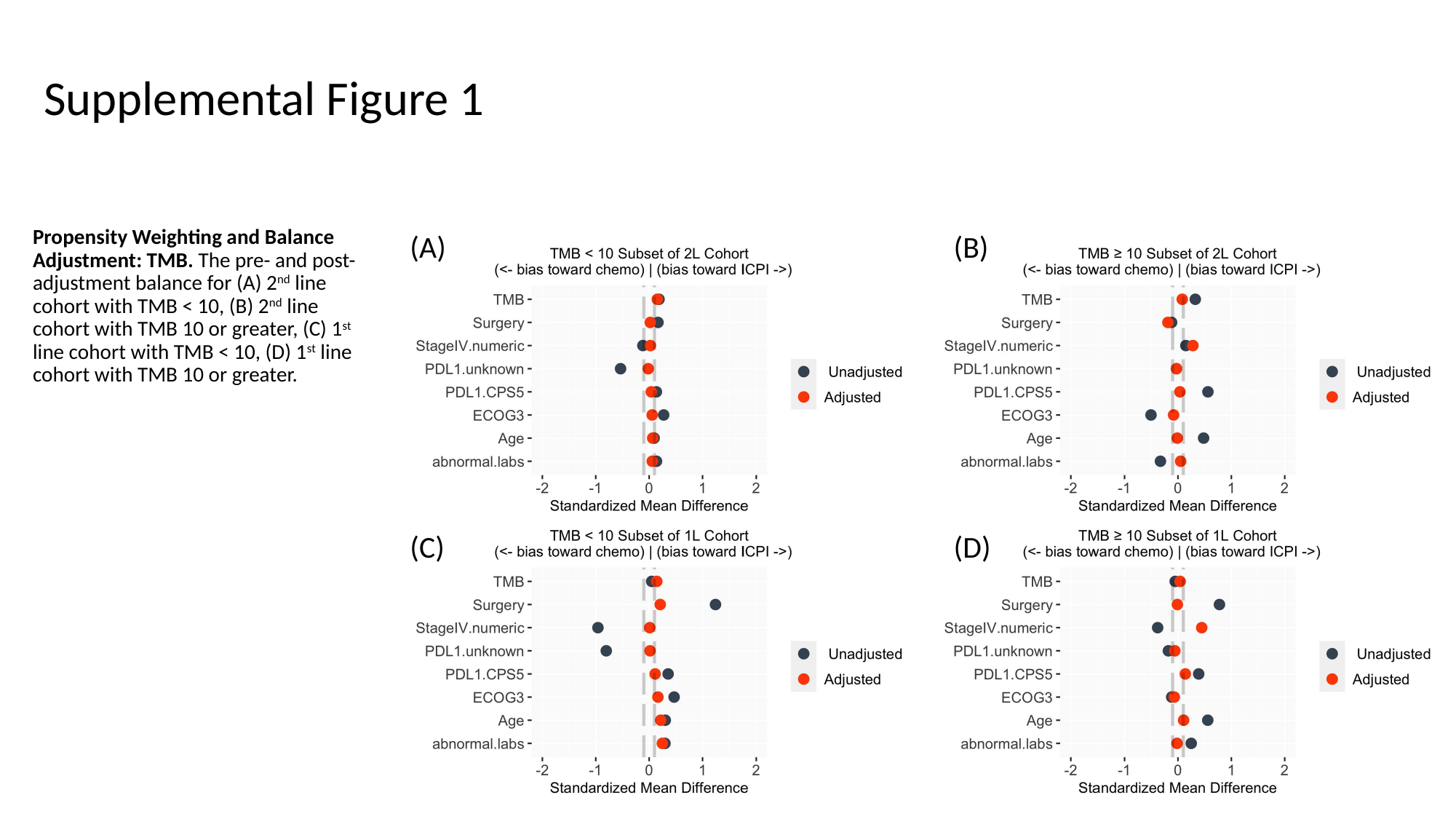

# Supplemental Figure 1
(A)
(B)
Propensity Weighting and Balance Adjustment: TMB. The pre- and post-adjustment balance for (A) 2nd line cohort with TMB < 10, (B) 2nd line cohort with TMB 10 or greater, (C) 1st line cohort with TMB < 10, (D) 1st line cohort with TMB 10 or greater.
(C)
(D)
