# Supplementary material for: Real-world Validation of TMB and Microsatellite Instability as Predictive Biomarkers of Immune Checkpoint Inhibitor Effectiveness in Advanced Gastroesophageal Cancer
Source: Cancer Res Commun. 2022 Sep 21;2(9):1037–48. doi: 10.1158/2767-9764.CRC-22-0161 (PMC10010289; doi:10.1158/2767-9764.CRC-22-0161)
Supplement: Figure S2 — 2nd line treatment-TMB interaction models from Figure 2. The (A) TTNT and (B) OS interaction models are shown for for propensity adjusted analyses in Figure 2. [file crc-22-0161-s10.pptx]

## Slide 1
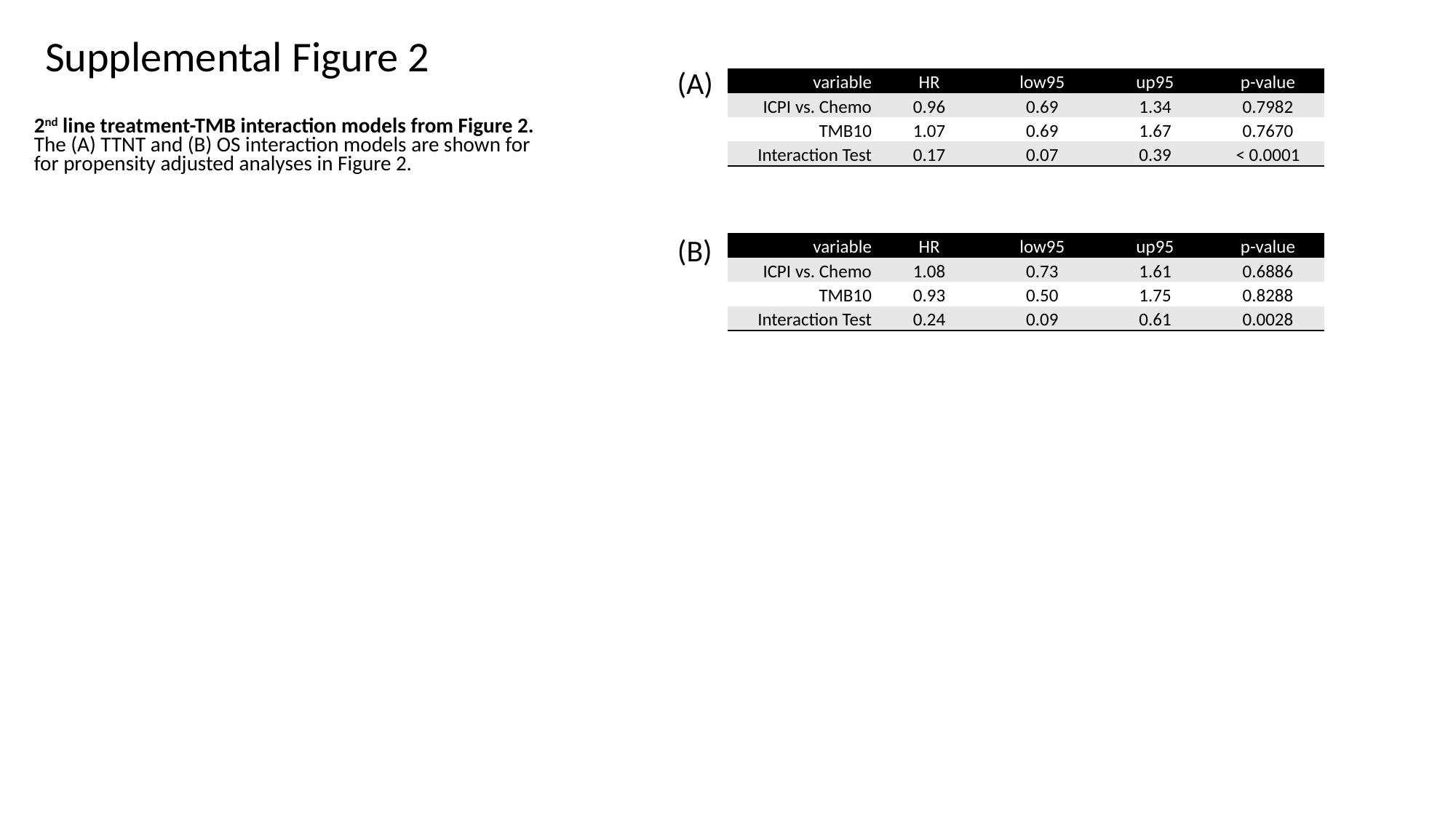

# Supplemental Figure 2
(A)
| variable | HR | low95 | up95 | p-value |
| --- | --- | --- | --- | --- |
| ICPI vs. Chemo | 0.96 | 0.69 | 1.34 | 0.7982 |
| TMB10 | 1.07 | 0.69 | 1.67 | 0.7670 |
| Interaction Test | 0.17 | 0.07 | 0.39 | < 0.0001 |
2nd line treatment-TMB interaction models from Figure 2. The (A) TTNT and (B) OS interaction models are shown for for propensity adjusted analyses in Figure 2.
(B)
| variable | HR | low95 | up95 | p-value |
| --- | --- | --- | --- | --- |
| ICPI vs. Chemo | 1.08 | 0.73 | 1.61 | 0.6886 |
| TMB10 | 0.93 | 0.50 | 1.75 | 0.8288 |
| Interaction Test | 0.24 | 0.09 | 0.61 | 0.0028 |
