# Supplementary material for: Real-world Validation of TMB and Microsatellite Instability as Predictive Biomarkers of Immune Checkpoint Inhibitor Effectiveness in Advanced Gastroesophageal Cancer
Source: Cancer Res Commun. 2022 Sep 21;2(9):1037–48. doi: 10.1158/2767-9764.CRC-22-0161 (PMC10010289; doi:10.1158/2767-9764.CRC-22-0161)
Supplement: Figure S3 — Unadjusted 2nd line treatment-TMB interaction models from Supplemental Figure 2. The (A) TTNT and (B) OS interaction models are shown for for propensity adjusted analyses in Supplemental Figure 2. [file crc-22-0161-s11.pptx]

## Slide 1
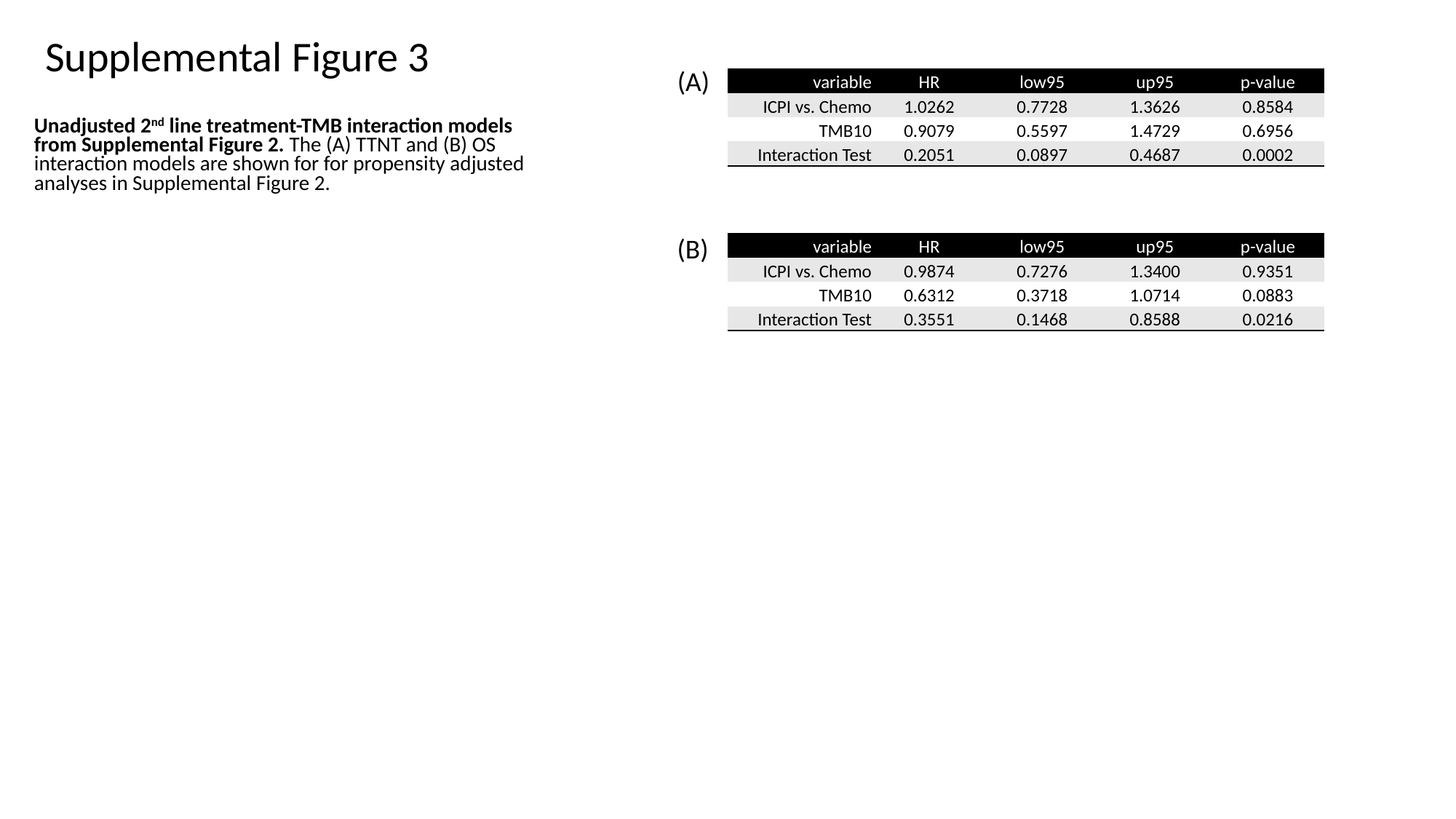

# Supplemental Figure 3
(A)
| variable | HR | low95 | up95 | p-value |
| --- | --- | --- | --- | --- |
| ICPI vs. Chemo | 1.0262 | 0.7728 | 1.3626 | 0.8584 |
| TMB10 | 0.9079 | 0.5597 | 1.4729 | 0.6956 |
| Interaction Test | 0.2051 | 0.0897 | 0.4687 | 0.0002 |
Unadjusted 2nd line treatment-TMB interaction models from Supplemental Figure 2. The (A) TTNT and (B) OS interaction models are shown for for propensity adjusted analyses in Supplemental Figure 2.
(B)
| variable | HR | low95 | up95 | p-value |
| --- | --- | --- | --- | --- |
| ICPI vs. Chemo | 0.9874 | 0.7276 | 1.3400 | 0.9351 |
| TMB10 | 0.6312 | 0.3718 | 1.0714 | 0.0883 |
| Interaction Test | 0.3551 | 0.1468 | 0.8588 | 0.0216 |
