# Supplementary material for: Real-world Validation of TMB and Microsatellite Instability as Predictive Biomarkers of Immune Checkpoint Inhibitor Effectiveness in Advanced Gastroesophageal Cancer
Source: Cancer Res Commun. 2022 Sep 21;2(9):1037–48. doi: 10.1158/2767-9764.CRC-22-0161 (PMC10010289; doi:10.1158/2767-9764.CRC-22-0161)
Supplement: Figure S4 — Unadjusted for known treatment assignment imbalances, patients receiving 2nd line ICPI vs. chemotherapy have more favorable outcomes when TMB ≥ 10 but not TMB < 10. Kaplan-Meier curves are unadjusted for imbalances. TTNT is shown by drug class for (A) TMB < 10, and (B) TMB ≥ 10. OS is shown by drug class for (C) TMB < 10, and (D) TMB ≥ 10. X-axis is truncated at 36 months. Overall survival estimates are left truncated (see Methods) with at-risk tables adjusted accordingly. Interaction models can be found in Supplemental Figure 8. [file crc-22-0161-s12.pptx]

## Slide 1
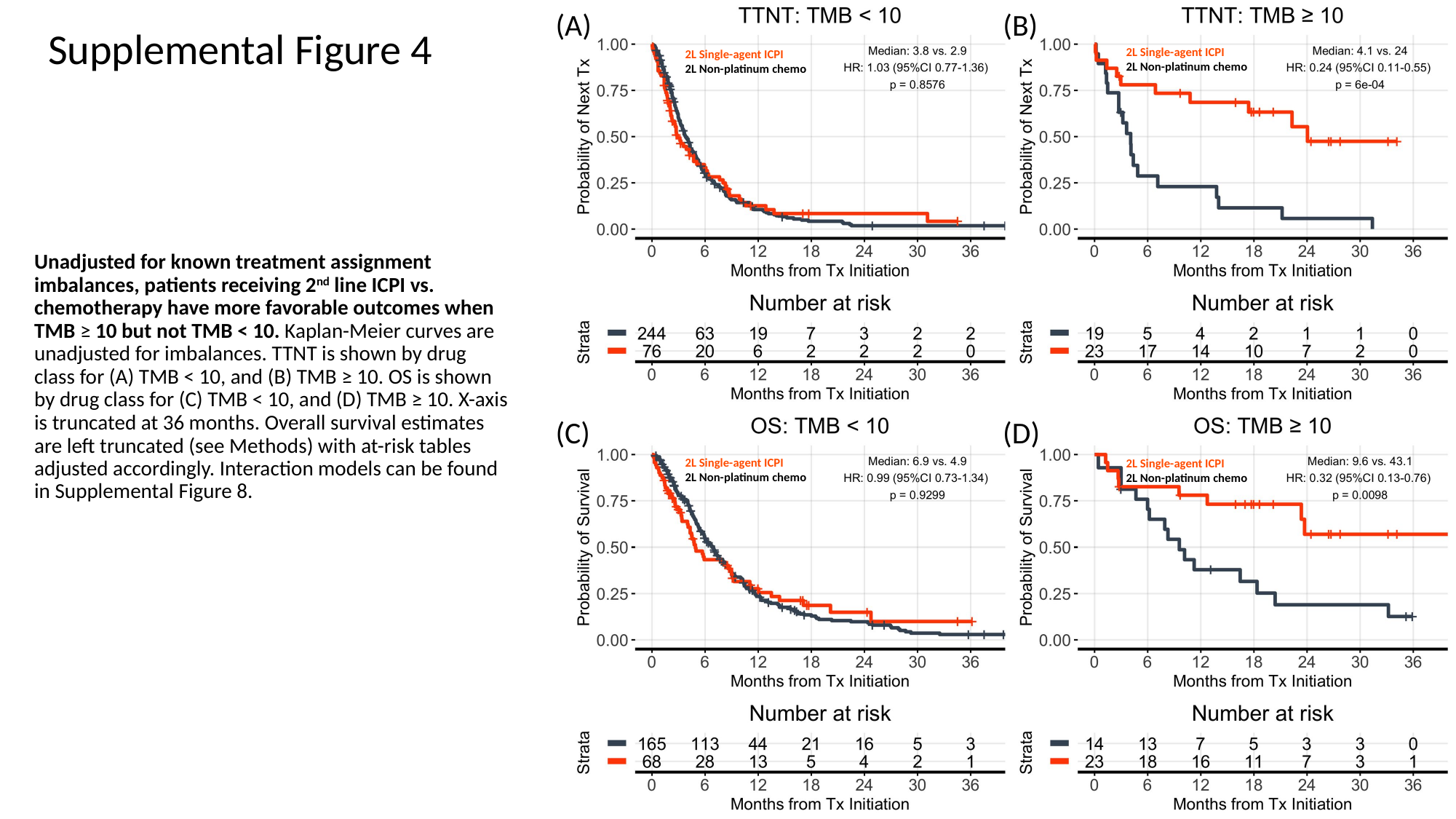

(A)
(B)
# Supplemental Figure 4
2L Single-agent ICPI
2L Non-platinum chemo
2L Single-agent ICPI
2L Non-platinum chemo
Unadjusted for known treatment assignment imbalances, patients receiving 2nd line ICPI vs. chemotherapy have more favorable outcomes when TMB ≥ 10 but not TMB < 10. Kaplan-Meier curves are unadjusted for imbalances. TTNT is shown by drug class for (A) TMB < 10, and (B) TMB ≥ 10. OS is shown by drug class for (C) TMB < 10, and (D) TMB ≥ 10. X-axis is truncated at 36 months. Overall survival estimates are left truncated (see Methods) with at-risk tables adjusted accordingly. Interaction models can be found in Supplemental Figure 8.
(C)
(D)
2L Single-agent ICPI
2L Non-platinum chemo
2L Single-agent ICPI
2L Non-platinum chemo
