# Supplementary material for: Real-world Validation of TMB and Microsatellite Instability as Predictive Biomarkers of Immune Checkpoint Inhibitor Effectiveness in Advanced Gastroesophageal Cancer
Source: Cancer Res Commun. 2022 Sep 21;2(9):1037–48. doi: 10.1158/2767-9764.CRC-22-0161 (PMC10010289; doi:10.1158/2767-9764.CRC-22-0161)
Supplement: Figure S5 — Sequential TTNT treatment-TMB interaction model from Figure 3. The treatment interaction model from Figure 3C is shown numerically. [file crc-22-0161-s13.pptx]

## Slide 1
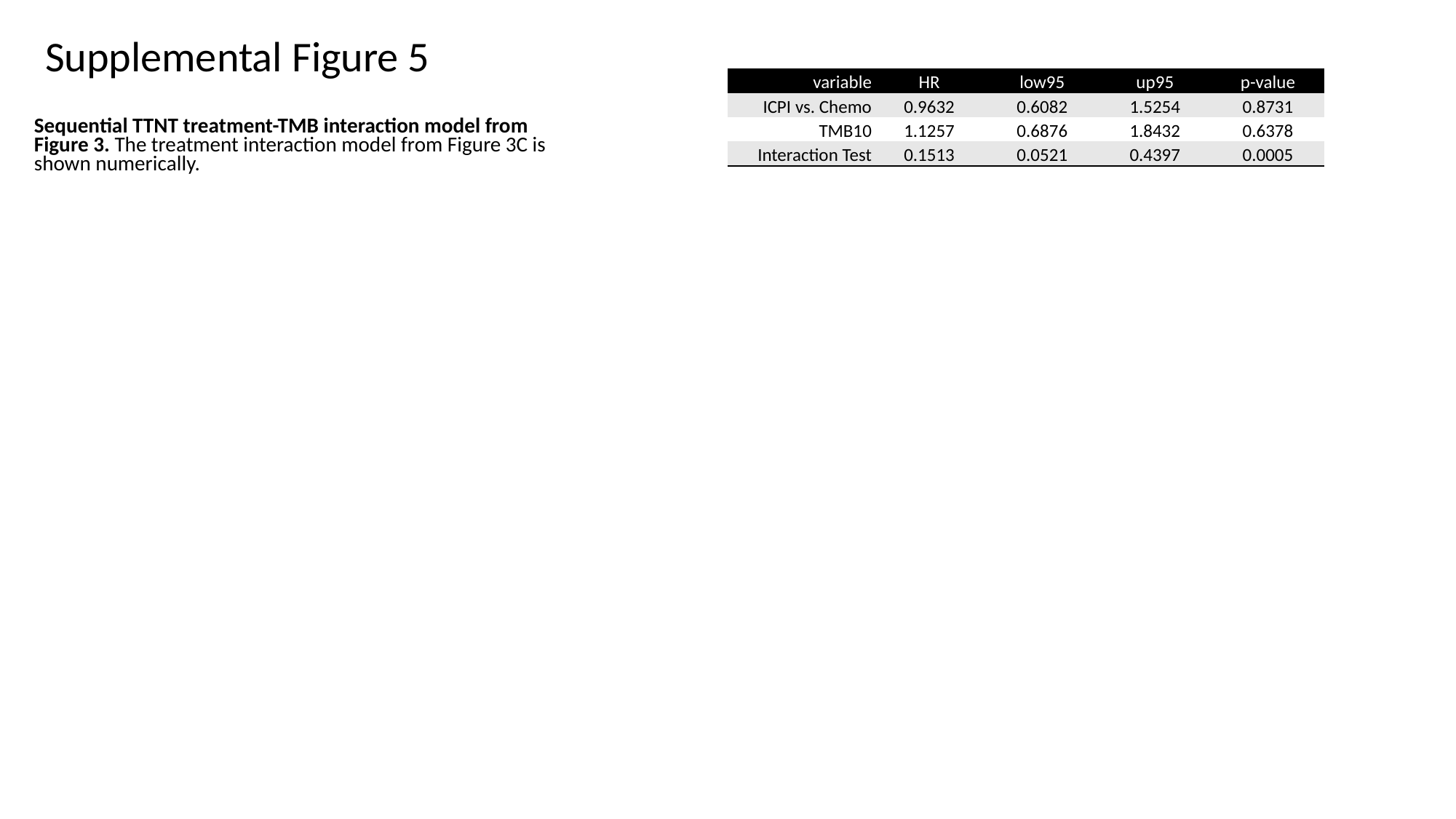

# Supplemental Figure 5
| variable | HR | low95 | up95 | p-value |
| --- | --- | --- | --- | --- |
| ICPI vs. Chemo | 0.9632 | 0.6082 | 1.5254 | 0.8731 |
| TMB10 | 1.1257 | 0.6876 | 1.8432 | 0.6378 |
| Interaction Test | 0.1513 | 0.0521 | 0.4397 | 0.0005 |
Sequential TTNT treatment-TMB interaction model from Figure 3. The treatment interaction model from Figure 3C is shown numerically.
