# Supplementary material for: Real-world Validation of TMB and Microsatellite Instability as Predictive Biomarkers of Immune Checkpoint Inhibitor Effectiveness in Advanced Gastroesophageal Cancer
Source: Cancer Res Commun. 2022 Sep 21;2(9):1037–48. doi: 10.1158/2767-9764.CRC-22-0161 (PMC10010289; doi:10.1158/2767-9764.CRC-22-0161)
Supplement: Figure S6 — Unadjusted 1st line treatment-TMB interaction models from Supplemental Figure 3. The (A) TTNT and (B) OS interaction models are shown for for propensity adjusted analyses in Supplemental Figure 3. [file crc-22-0161-s14.pptx]

## Slide 1
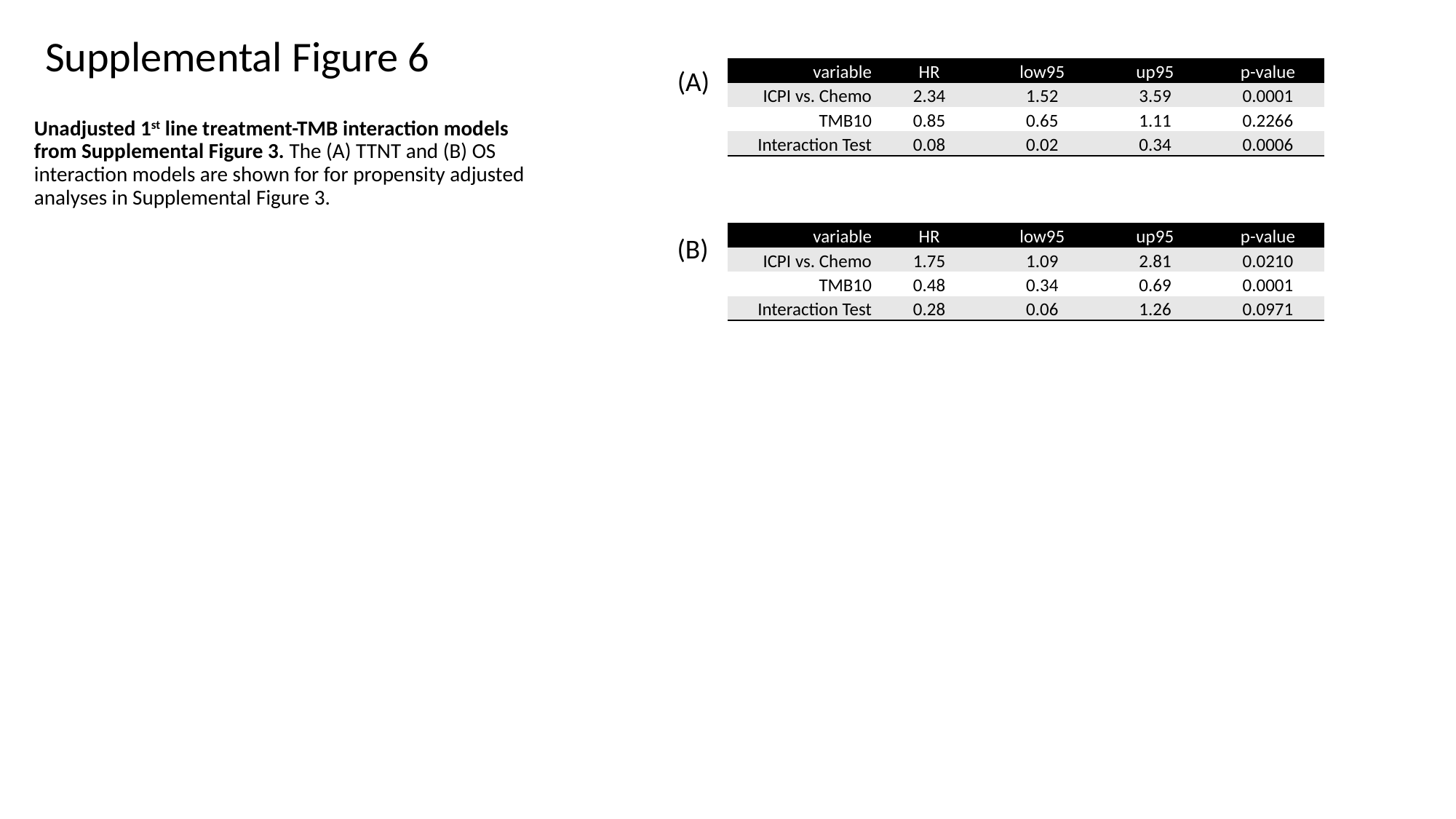

# Supplemental Figure 6
(A)
| variable | HR | low95 | up95 | p-value |
| --- | --- | --- | --- | --- |
| ICPI vs. Chemo | 2.34 | 1.52 | 3.59 | 0.0001 |
| TMB10 | 0.85 | 0.65 | 1.11 | 0.2266 |
| Interaction Test | 0.08 | 0.02 | 0.34 | 0.0006 |
Unadjusted 1st line treatment-TMB interaction models from Supplemental Figure 3. The (A) TTNT and (B) OS interaction models are shown for for propensity adjusted analyses in Supplemental Figure 3.
| variable | HR | low95 | up95 | p-value |
| --- | --- | --- | --- | --- |
| ICPI vs. Chemo | 1.75 | 1.09 | 2.81 | 0.0210 |
| TMB10 | 0.48 | 0.34 | 0.69 | 0.0001 |
| Interaction Test | 0.28 | 0.06 | 1.26 | 0.0971 |
(B)
