# Supplementary material for: Real-world Validation of TMB and Microsatellite Instability as Predictive Biomarkers of Immune Checkpoint Inhibitor Effectiveness in Advanced Gastroesophageal Cancer
Source: Cancer Res Commun. 2022 Sep 21;2(9):1037–48. doi: 10.1158/2767-9764.CRC-22-0161 (PMC10010289; doi:10.1158/2767-9764.CRC-22-0161)
Supplement: Figure S7 — Discriminatory Power Comparison of MSI and TMB. The concordance indexes from univariable Cox PH models containing NGS-based MSI assessment, TMB, or the two in a combined multivariable Cox PH model is shown with (A) TTNT and (B) OS. Error bars indicate standard error. [file crc-22-0161-s15.pptx]

## Slide 1
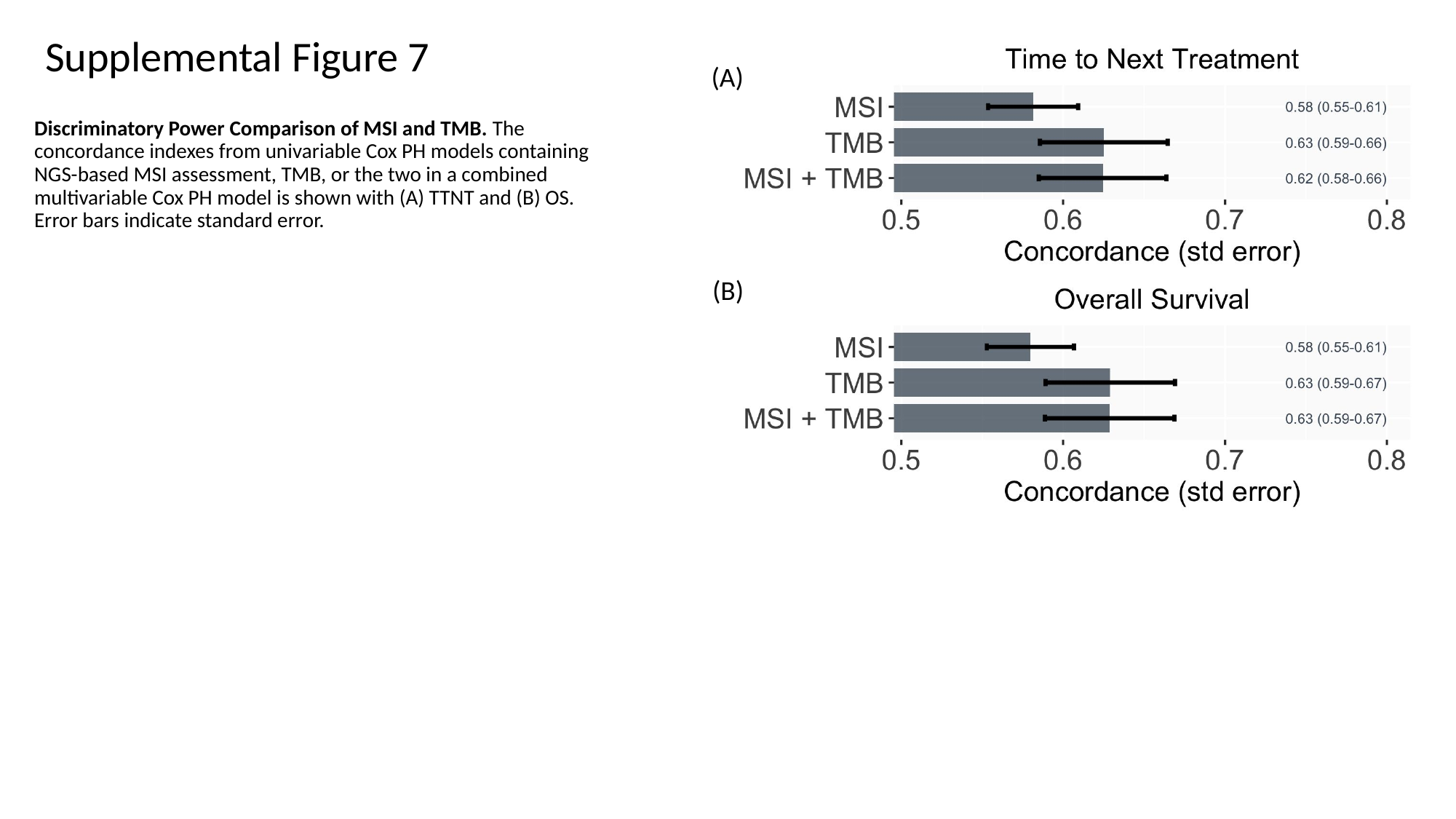

# Supplemental Figure 7
(A)
Discriminatory Power Comparison of MSI and TMB. The concordance indexes from univariable Cox PH models containing NGS-based MSI assessment, TMB, or the two in a combined multivariable Cox PH model is shown with (A) TTNT and (B) OS. Error bars indicate standard error.
(B)
