# Supplementary material for: Real-world Validation of TMB and Microsatellite Instability as Predictive Biomarkers of Immune Checkpoint Inhibitor Effectiveness in Advanced Gastroesophageal Cancer
Source: Cancer Res Commun. 2022 Sep 21;2(9):1037–48. doi: 10.1158/2767-9764.CRC-22-0161 (PMC10010289; doi:10.1158/2767-9764.CRC-22-0161)
Supplement: Figure S8 — TMB ≥ 10 treatment predictive associations is not approximated by PD-L1 CPS ≥ 10. Cox PH models containing treatment interaction terms for both TMB ≥ 10 and PD-L1 CPS ≥ 10 are shown numerically (A) 2nd line cohort TTNT, (B) 2nd line cohort OS, and (C) sequential cohort TTNT. [file crc-22-0161-s16.pptx]

## Slide 1
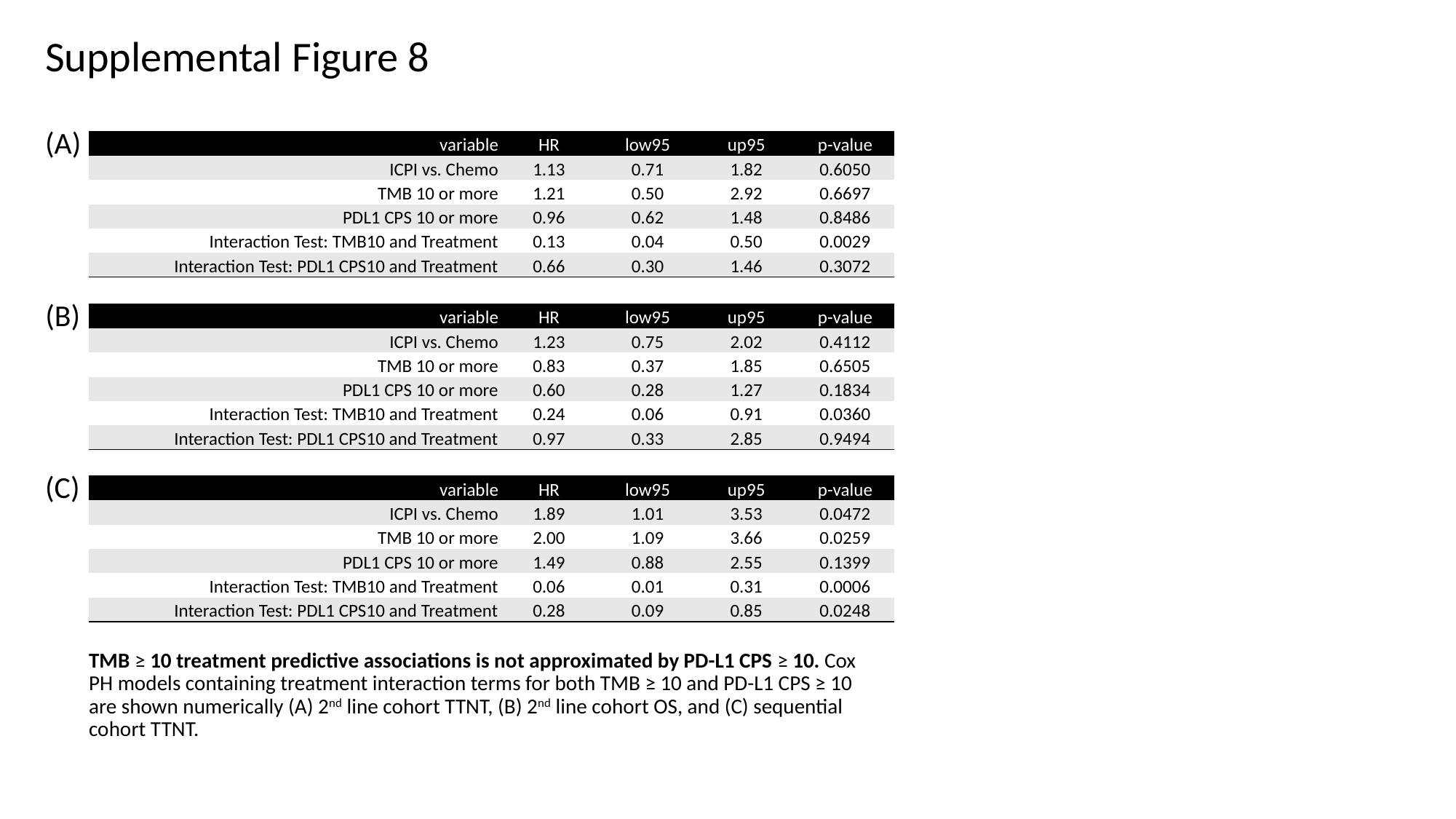

# Supplemental Figure 8
(A)
| variable | HR | low95 | up95 | p-value |
| --- | --- | --- | --- | --- |
| ICPI vs. Chemo | 1.13 | 0.71 | 1.82 | 0.6050 |
| TMB 10 or more | 1.21 | 0.50 | 2.92 | 0.6697 |
| PDL1 CPS 10 or more | 0.96 | 0.62 | 1.48 | 0.8486 |
| Interaction Test: TMB10 and Treatment | 0.13 | 0.04 | 0.50 | 0.0029 |
| Interaction Test: PDL1 CPS10 and Treatment | 0.66 | 0.30 | 1.46 | 0.3072 |
(B)
| variable | HR | low95 | up95 | p-value |
| --- | --- | --- | --- | --- |
| ICPI vs. Chemo | 1.23 | 0.75 | 2.02 | 0.4112 |
| TMB 10 or more | 0.83 | 0.37 | 1.85 | 0.6505 |
| PDL1 CPS 10 or more | 0.60 | 0.28 | 1.27 | 0.1834 |
| Interaction Test: TMB10 and Treatment | 0.24 | 0.06 | 0.91 | 0.0360 |
| Interaction Test: PDL1 CPS10 and Treatment | 0.97 | 0.33 | 2.85 | 0.9494 |
(C)
| variable | HR | low95 | up95 | p-value |
| --- | --- | --- | --- | --- |
| ICPI vs. Chemo | 1.89 | 1.01 | 3.53 | 0.0472 |
| TMB 10 or more | 2.00 | 1.09 | 3.66 | 0.0259 |
| PDL1 CPS 10 or more | 1.49 | 0.88 | 2.55 | 0.1399 |
| Interaction Test: TMB10 and Treatment | 0.06 | 0.01 | 0.31 | 0.0006 |
| Interaction Test: PDL1 CPS10 and Treatment | 0.28 | 0.09 | 0.85 | 0.0248 |
TMB ≥ 10 treatment predictive associations is not approximated by PD-L1 CPS ≥ 10. Cox PH models containing treatment interaction terms for both TMB ≥ 10 and PD-L1 CPS ≥ 10 are shown numerically (A) 2nd line cohort TTNT, (B) 2nd line cohort OS, and (C) sequential cohort TTNT.
