# Supplementary material for: Real-world Validation of TMB and Microsatellite Instability as Predictive Biomarkers of Immune Checkpoint Inhibitor Effectiveness in Advanced Gastroesophageal Cancer
Source: Cancer Res Commun. 2022 Sep 21;2(9):1037–48. doi: 10.1158/2767-9764.CRC-22-0161 (PMC10010289; doi:10.1158/2767-9764.CRC-22-0161)
Supplement: Figure S9 — 1st line treatment-TMB interaction models from Figure 5. The (A) TTNT and (B) OS interaction models are shown for for propensity adjusted analyses in Figure 5. [file crc-22-0161-s17.pptx]

## Slide 1
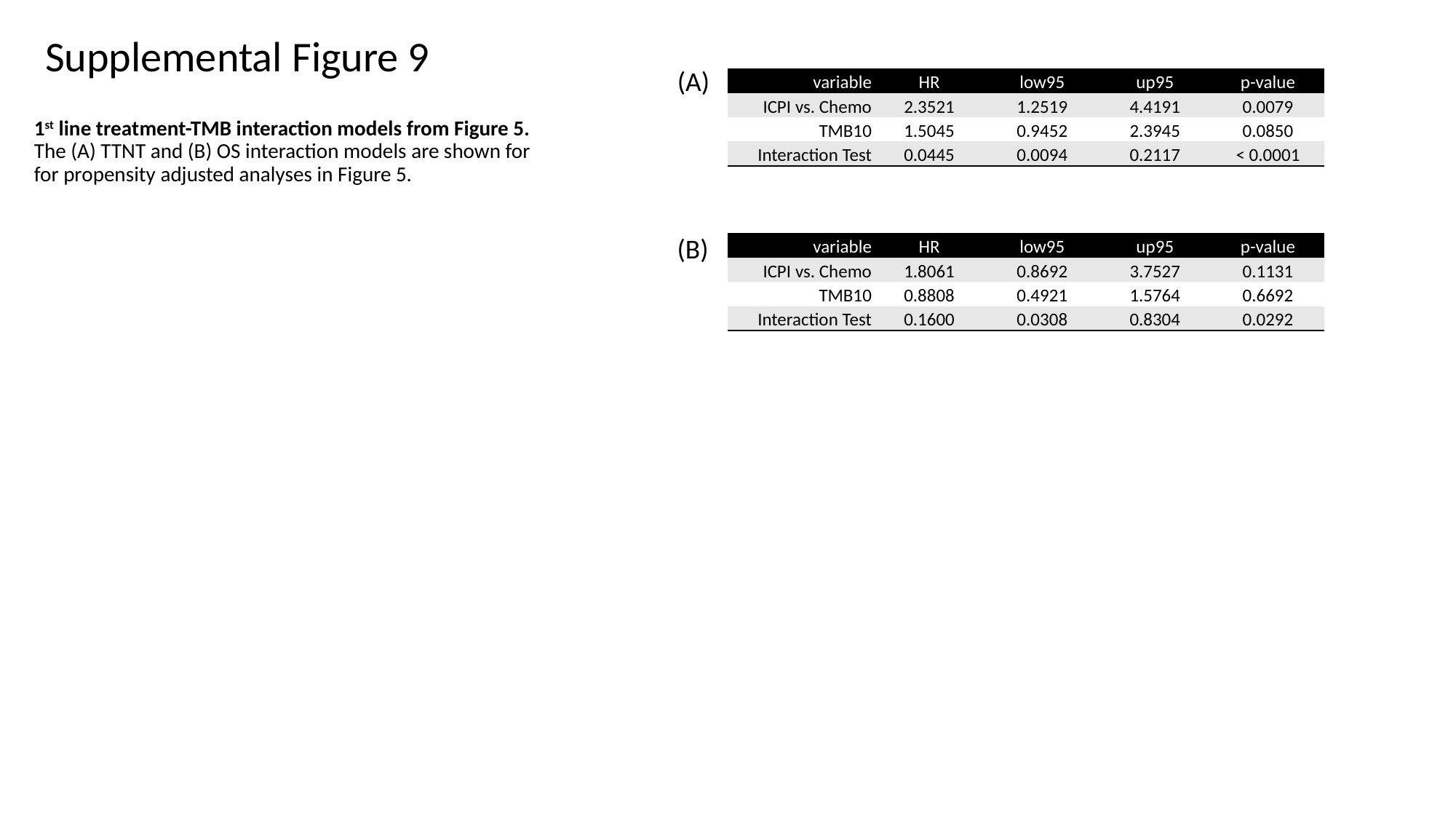

# Supplemental Figure 9
(A)
| variable | HR | low95 | up95 | p-value |
| --- | --- | --- | --- | --- |
| ICPI vs. Chemo | 2.3521 | 1.2519 | 4.4191 | 0.0079 |
| TMB10 | 1.5045 | 0.9452 | 2.3945 | 0.0850 |
| Interaction Test | 0.0445 | 0.0094 | 0.2117 | < 0.0001 |
1st line treatment-TMB interaction models from Figure 5. The (A) TTNT and (B) OS interaction models are shown for for propensity adjusted analyses in Figure 5.
(B)
| variable | HR | low95 | up95 | p-value |
| --- | --- | --- | --- | --- |
| ICPI vs. Chemo | 1.8061 | 0.8692 | 3.7527 | 0.1131 |
| TMB10 | 0.8808 | 0.4921 | 1.5764 | 0.6692 |
| Interaction Test | 0.1600 | 0.0308 | 0.8304 | 0.0292 |
